# Synthesis, Structure, and Biologic Activity of Some Copper, Nickel, Cobalt, and Zinc Complexes with 2-Formylpyridine *N*^4^-Allylthiosemicarbazone

**DOI:** 10.1155/2022/2705332

**Published:** 2022-05-25

**Authors:** Vasilii Graur, Yurii Chumakov, Olga Garbuz, Christelle Hureau, Victor Tsapkov, Aurelian Gulea

**Affiliations:** ^1^Laboratory of Advanced Materials in Biofarmaceutics and Technics, Moldova State University, 60 Mateevici Street, MD 2009, Chisinau, Moldova; ^2^Institute of Applied Physics, 5 Academiei Street, MD 2028, Chisinau, Moldova; ^3^Laboratoire de Chimie de Coordination, 205 Route de Narbonne 31077 Toulouse Cedex 04, Toulouse, France

## Abstract

A series of zinc(II) ([Zn(H_2_O)(L)Cl] (**1**)), copper (II) ([Cu(L)Cl] (**2**), [Cu(L)Br] (**3**), [Cu_2_(L)_2_(CH_3_COO)_2_]·4H_2_O (**4**)), nickel(II) ([Ni(*HL*)_2_]Cl_2_·H_2_O (**5**)), and cobalt(III) ([Co(L)_2_]Cl (**6**)) complexes were obtained with 2-formylpyridine *N*^4^-allylthiosemicarbazone (*HL*). In addition another two thiosemicarbazones (3-formylpyridine *N*^4^-allylthiosemicarbazone (*HL*^a^) and 4-formylpyridine *N*^4^-allylthiosemicarbazone (*HL*^b^)) have been obtained. The synthesized thiosemicarbazones have been studied using ^1^H and ^13^C NMR spectroscopy, IR spectroscopy, and X-ray diffraction analysis. The composition and structure of complexes were studied using elemental analysis, IR and UV-Vis spectroscopies, molar conductivity, and magnetic susceptibility measurements. Single crystal X-ray diffraction analysis elucidated the structure of thiosemicarbazones *HL*, *HL*^a^, and *HL*^b^, as well as complexes **4** and **5**. The antiproliferative properties of these compounds toward a series of cancer cell lines (HL-60, HeLa, BxPC-3, RD) and a normal cell line (MDCK) have been investigated. The nickel complex shows high selectivity (SI > 1000) toward HL-60 cell line and is the least toxic. The zinc complex shows the highest selectivity toward RD cell line (SI = 640). The copper complexes (**2**–**4**) are the most active molecular inhibitors of proliferation of cancer cells, but exhibit not such a high selectivity and are significantly more toxic. Zinc and copper complexes manifest high antibacterial activity. It was found that calculated at B3LYP level of theory different reactivity descriptors of studied compounds strongly correlate with their biological activity.

## 1. Introduction

The steady increase in the number of diseases poses a challenge for modern chemistry in the search for new biologically active substances that will allow to treat these diseases and enhance the duration and quality of life [1–6]. The number of cancers continues to rise [7]. Therefore, a great attention is paid to the new anticancer compounds [8, 9]. Because the main disadvantage of substances, which are used for cancer treatment, is their general toxicity for the human organism, special attention should be paid to the study of selectivity of antiproliferative activity and the toxicity of these substances. The expansion of the arsenal of such substances will allow in the future to personally select the active substance for the treatment of this disease, which should increase the effectiveness of treatment [10].

Thiosemicarbazones and biometal complexes with them represent one of the classes of compounds that are widely studied in this direction [11–15].

Among thiosemicarbazones of aliphatic [16–18], aromatic [19–21], and heteroaromatic [22–24] aldehydes and ketones, derivatives of heteroaromatic carbonyl compounds, especially thiosemicarbazones of *α*-*N*-heteroaromatic aldehydes and ketones, usually exhibit the most pronounced biological properties [25, 26]. Thiosemicarbazones of *α*-*N*-heteroaromatic aldehydes and ketones most often act as tridentate ligands with an NNS-set of donor atoms due to the position of the nitrogen atom in the carbonyl moiety, thus forming two five-membered metallocycles [27]. Usually, the introduction of substituents in the fourth position of the thiosemicarbazide moiety leads to a significant increase in biological properties [28]. Some of these compounds (3-amino-2-pyridinecarboxaldehyde thiosemicarbazone (triapine), di-2-pyridylketone 4-cyclohexyl-4-methylthiosemicarbazone) are undergoing pre-clinical and clinical trials [29, 30].

Copper complexes are the most studied among the transition metal complexes with thiosemicarbazones from the biological point of view. In many cases, the coordination of thiosemicarbazones to the copper(II) ion leads to the greatest enhancement of anticancer and antimicrobial activity [31–34]. Despite the fact that the selectivity of the described copper complexes in many cases exceeds the selectivity of anticancer drugs such as doxorubicin and cisplatin, in most cases the selectivity index does not exceed 10 [35]. Among *N*^4^-substituted thiosemicarbazones more attention is paid to *N*^4^-alkyl, *N*^4^-arylthiosemicarbazones. *N*^4^-allylthiosemicarbazones are described less frequently. However, *N*^4^-allylthiosemicarbazones exhibit high biological activity and are comparatively more soluble in water. Based on this, it is important to continue the systematic study of thiosemicarbazones and biometal complexes with them in order to identify new substances with high anticancer, antimicrobial, and antifungal properties, which at the same time will be free from such a significant drawback as high toxicity.

**Scheme 1:**
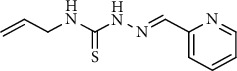
2-Formylpyridine *N*^4^-allylthiosemicarbazone (*HL*).

In order to find such substances, the present study describes the chemical synthesis and characterization of Zn(II), Cu(II), Ni(II), and Co(III) complexes with 2-formylpyridine *N*^4^-allylthiosemicarbazone (Scheme 1), *in vitro* biological evaluation, and *in vivo* toxicity of these substances.

## 2. Materials and Methods

### 2.1. Materials

All the reagents used were chemically pure. 3*d*-Metal salts Zn(CH_3_COO)_2_·2H_2_O, CuCl_2_·2H_2_O, CuBr_2,_ Cu(CH_3_COO)_2_·H_2_O, NiCl_2_·6H_2_O, and CoCl_2_·6H_2_O (Merck, Darmstadt, Germany) were used as supplied. 2-, 3- and 4-formylpyridines, nystatin, furacillinum, doxorubicin, and trolox were used as received (Sigma-Aldrich, Munich, Germany). *N*^4^-allyl-3-thiosemicarbazide was prepared from allylisothiocyanate by reaction with hydrazine hydrate [6, 36, 37]. The solvents were purified and dried according to standard procedures [38].

### 2.2. Synthesis of the *N*^4^-Allylthiosemicarbazones

#### 2.2.1. Synthesis of the 2-Formylpyridine *N*^4^-Allylthiosemicarbazone (*HL*)

**Scheme 2:**
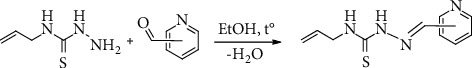
Synthesis of 2-,3-, and 4-formylpyridine *N*^4^-allylthiosemicarbazones.

2-Formylpyridine *N*^4^-allylthiosemicarbazone (*HL*) has been synthesized as described by Zeglis B. M. et al. [39] by reaction between *N*^4^-allyl-3-thiosemicarbazide and 2-formylpyridine in a 1 : 1 molar ratio (Scheme 2). The solution of *N*^4^-allyl-3-thiosemicarbazide (1.31 g, 10 mmol) was prepared upon stirring and heating it in 30 mL of ethanol (96% v/v). 2-Formylpyridine (1.07 g, 10 mmol) was then added to the obtained colorless solution. As a result, a yellow solution was obtained. It was heated (80°C) with constant stirring for 1 h. A pale yellow precipitate has formed upon cooling of the reaction mixture. It was separated by filtration, washed with cold ethanol, and dried *in vacuo*.

Pale yellow solid. Yield: 89%; m.p.: 157–159°C; FW: 220.29 g/mol; Anal. Calc. for C_10_H_12_N_4_S : C, 54.52; H, 5.49; N, 25.43; S, 14.56%. Found: C, 54.40; H, 5.33; N, 25.68; S, 14.32%. Main IR peaks (cm^−1^) :  *ν* (NH) 3365, 3140; *ν*(C = C allyl) 1642, *ν* (C = N^1^) 1602, *ν* (С = N_pyr_) 1583, and *ν* (C = S) 1313.

^1^H NMR (acetone-d_6_; *δ*, ppm) (Figure S1): 10.78 (br, 1H, NH); 8.60 (s, 1H, CH aromatic); 8.59 (br, 1H, NH); 8.23 (s, 1H, CH = N^1^); 8.11 (d, CH aromatic); 7.80 (t, 1H, CH aromatic); 7.36 (t, 1H, CH aromatic); 5.99 (m, 1H, CH from allyl moiety); 5.17 (m, 2H, CH_2_ = C from allyl moiety); and 4.37 (m, 2H, CH_2_-N).

^13^C NMR (acetone-d_6_; *δ*, ppm) (Figure S2): 178.73 (C = S); 153.64, 149.56, 136.32, 123.98, 119.95 (C aromatic); 142.43 (CH = N^1^); 134.60 (CH from allyl moiety); 115.36 (CH_2_ = from allyl moiety); and 46.28 (CH_2_-N).

#### 2.2.2. Synthesis of the 3-Formylpyridine *N*^4^-Allylthiosemicarbazone (*HL*^a^)

The thiosemicarbazone *HL*^a^ was obtained similarly to *HL*. *N*^4^-Allyl-3-thiosemicarbazide (1.31 g, 10 mmol) was dissolved in 30 mL of ethanol (96% v/v). After that 3-formylpyridine (1.07 g, 10 mmol) was added to the reaction mixture. The obtained homogeneous system was heated (80°C) with constant stirring for 1 h. A pale yellow precipitate has formed upon cooling of the reaction mixture. It was separated by filtration, washed with cold ethanol, and dried *in vacuo* (Scheme 2).

Pale yellow solid. Yield: 87%; m.p.: 180–182°C; FW: 220.29 g/mol; Anal. Calc. for C_10_H_12_N_4_S : C, 54.52; H, 5.49; N, 25.43; S, 14.56%. Found: C, 58.71; H, 5.44; N, 25.22; S, 14.61%. Main IR peaks (cm^−1^) : *ν* (NH) 3351, 3143; *ν* (C = C allyl) 1644; *ν*(C = N^1^) 1611; *ν* (С = N_pyr_) 1593; *ν* (C = S) 1303.

^1^H NMR (DMSO-d_6_; *δ*, ppm) (Figure S3): 11.70 (br, 1H, NH); 8.97 (s, 1H, CH aromatic); 8.85 (br, 1H, NH); 8.57 (d, 1H, aromatic); 8.26 (d, 1H, CH aromatic); 8.08 (s, 1H, CH = N); 7.44 (m, 1H, CH aromatic); 5.91 (m, 1H, CH from allyl moiety); 5.13 (m, 2H, CH_2_ = C from allyl moiety); 4.23 (m, 2H, CH_2_-N).

^13^C NMR (DMSO-d_6_; *δ*, ppm) (Figure S4): 177.84 (C = S); 150.73, 149.18, 135.44, 130.67, 124.20 (C aromatic); 139.52 (CH = N^1^); 134.39 (CH from allyl moiety); 116.03 (CH_2_ = from allyl moiety); 46.29 (CH_2_-N).

#### 2.2.3. Synthesis of the 4-Formylpyridine *N*^4^-Allylthiosemicarbazone (*HL*^b^)

The thiosemicarbazone *HL*^b^ was obtained similarly to *HL*^a^ using *N*^4^-allyl-3-thiosemicarbazide (1.31 g, 10 mmol) and 4-formylpyridine (1.07 g, 10 mmol) instead of 3-formylpyridine (Scheme 2).

Pale yellow solid. Yield: 86%; m.p.: 163–165°C; FW:  220.29 g/mol; Anal. Calc. for C_10_H_12_N_4_S : C, 54.52; H, 5.49; N, 25.43; S, 14.56%. Found: C, 54.37; H, 5.48; N, 25.54; S, 14.40%. Main IR peaks (cm^−1^): *ν* (NH) 3223, 3125; *ν* (C = C allyl) 1642; *ν*(C = N^1^) 1603, *ν*(С = N_pyr_) 1593; *ν*(C = S) 1305.

^1^H NMR (DMSO-d_6_; *δ*, ppm) (Figure S5): 11.82 (br, 1H, NH); 8.93 (br, 1H, NH); 8.61 (d, 2H, CH aromatic); 8.02 (s, 1H, CH = N); 7.79 (d, 2H, CH аром.); 5.91 (m, 1H, CH from allyl moiety); 5.14 (m, 2H, CH_2_ = C from allyl moiety); 4.23 (m, 2H, CH_2_-N).

^13^C NMR (DMSO-d_6_; *δ*, ppm) (Figure S6): 178.03 (C = S); 150.48, 141.95, 121.61 (C aromatic); 139.69 (CH = N); 135.28 (CH from allyl moiety); 116.12 (CH_2_ = from allyl moiety); 46.33 (CH_2_-N).

### 2.3. Synthesis of the Complexes **1**–**6**

The direct interaction of biometal salts with the ethanolic solutions of **3**-formylpyridine *N*^4^-allylthiosemicarbazone was used to obtain complexes **1**–**6**.

#### 2.3.1. Synthesis of the Complex [Zn(H_2_O)(L)Cl] (**1**)

2-Formylpyridine *N*^4^-allylthiosemicarbazone (*HL*) (0.220 g, 1 mmol) was dissolved in 25 mL of ethanol upon heating (55°C). After that, a sample of solid zinc chloride (ZnCl_2_) (0.136 g, 1 mmol) was added to the hot solution. The resultant mixture was stirred for 40 min at 55°C. The yellow precipitate of complex **1** was obtained upon cooling to room temperature. It was filtered out, washed with cold ethanol, and dried *in vacuo*.

Yellow solid. Yield: 74%; FW: 338.16 g/mol; Anal. Calc. for C_10_H_13_ClN_4_OSZn : C, 35.52; H, 3.87; Cl, 10.48; N, 16.57; S, 9.48; Zn, 19.34; Found: C, 35.36; H, 3.91; Cl, 10.54; N, 16.64; S, 9.73; Zn, 19.51. Main IR peaks (cm^−1^) : *ν* (NH) 3183, *ν*(C = C allyl) 1642, *ν*(C = N^2^) 1596, *ν*(C = N^1^) 1578, *ν*(С = N_pyr_) 1556, *ν*(C–S) 755. *μ*_eff_ (293 ± 1K): 0 *μ*_B_; *χ* (CH_3_OH): 56 Ω^−1^ cm^2^ mol^−1^.

#### 2.3.2. Synthesis of the Complex [Cu(L)Cl] (**2**)

The complex **2** has been obtained as described by Zeglis B.M. et al. [39]. Solid copper(II) chloride dihydrate (0.170 g; 1 mmol) was added to a hot ethanolic solution of 2-formylpyridine *N*^4^-allylthiosemicarbazone (*HL*) (0.220 g; 1 mmol). The resultant green solution was stirred and heated (55°C) for 40 min. Green precipitate was formed upon cooling to room temperature. It was filtered out, washed with cold ethanol, and dried *in vacuo*.

Green solid. Yield: 81%; FW: 318.29 g/mol; Anal. Calc. for C_10_H_11_ClCuN_4_S : C, 37.74; H, 3.48; Cl, 11.14; Cu, 19.97; N, 17.60; S, 10.07; Found: C, 37.87; H, 3.42; Cl, 11.30; Cu, 19.82; N, 17.41; S, 9.93; Main IR peaks (cm^−1^) :  *ν*(NH) 3188, *ν*(C = C allyl) 1637, *ν*(C = N^2^) 1588, *ν*(C = N^1^) 1567, *ν*(С = N_pyr_) 1560, *ν*(C–S) 765. *μ*_eff_ (293 ± 1K): 1.85 *μ*_B_; *χ* (CH_3_OH): 32 Ω^−1^ cm^2^ mol^−1^.

#### 2.3.3. Synthesis of the Complex [Cu(L)Br] (**3**)

The complex **3** was synthesized similarly to compound **1** using CuBr_2_ (0.223 g; 1 mmol) and *HL* (0.220 g; 1 mmol).

Green solid. Yield: 85%; FW: 362.74 g/mol; Anal. Calc. for C_10_H_11_BrCuN_4_S : C, 33.11; H, 3.06; Br, 22.03; Cu, 17.52; N, 15.45; S, 8.84; Found: C, 32.98; H, 3.13; Br, 21.88; Cu, 17.63; N, 15.54; S, 8.95; Main IR peaks (cm^−1^) : *ν* (NH) 3203, *ν*(C = C allyl) 1640, *ν*(C = N^2^) 1592, *ν*(C = N^1^) 1570, *ν*(С = N_pyr_) 1560, *ν*(C–S) 769. *μ*_eff_ (293 ± 1K): 1.81 *μ*_B_; *χ* (CH_3_OH): 52 Ω^−1^ cm^2^ mol^−1^.

#### 2.3.4. Synthesis of the Complex [Cu_2_(L)_2_(CH_3_COO)_2_]·4H_2_O (**4**)

The complex **4** was synthesized similarly to compound **1** using Cu(CH_3_COO)_2_·H_2_O (0.200 g; 1 mmol) and *HL* (0.220 g; 1 mmol).

Green solid. Yield: 82%; FW: 755.81 g/mol; Anal. Calc. for C_24_H_36_Cu_2_N_8_O_8_S_2_ : C, 38.14; H, 4.80; Cu, 16.82; N, 14.83; S, 8.48; Found: C, 38.27; H, 4.66; Cu, 16.93; N, 14.68; S, 8.57. Main IR peaks (cm^−1^): *ν*(NH) 3286, *ν*(C = C allyl) 1644, *ν*(C = N^2^) 1584, *ν*(C = N^1^) 1563, *ν*(*С* = N_pyr_) 1538, *ν*(C–S) 765. *μ*_eff_ (293 ± 1K): 1.25 *μ*_B_; *χ* (CH_3_OH): 34 Ω^−1^ cm^2^ mol^−1^.

#### 2.3.5. Synthesis of the Complex [Ni(*HL*)_2_]Cl_2_·H_2_O (5)

The complex **5** was synthesized similarly to compound **1**, but in this case NiCl_2_·6H_2_O (0.238 g; 1 mmol) and *HL* (0.440 g; 2 mmol) were taken in 1 : 2 molar ratio in 30 mL of ethanol.

Brown solid. Yield: 71%; FW: 588.20 g/mol; Anal. Calc. for C_20_H_26_Cl_2_N_8_NiOS_2_ : C, 40.84; H, 4.46; Cl, 12.05; N, 19.05; Ni, 9.98; S, 10.90; Found: C, 40.71; H, 4.52; Cl, 11.98; N, 18.94; Ni, 9.85; S, 10.81. Main IR peaks (cm^−1^) : *ν* (NH) 3207, 3170; *ν*(C = C allyl) 1643; *ν*(C = N^1^) 1588, *ν*(С = N_pyr_) 1566, *ν*(C = S) 1362. *μ*_eff_ (293 ± 1K): 2.84 *μ*_B_; *χ* (CH_3_OH): 158 Ω^−1^ cm^2^ mol^−1^.

#### 2.3.6. Synthesis of the Complex [Co(L)_2_]Cl (**6**)

The complex **6** was synthesized similarly to compound **5** using CoCl_2_·6H_2_O (0.238 g; 1 mmol) and *HL* (0.440 g; 2 mmol).

Brown solid. Yield: 83%; FW : 532.96 g/mol; Anal. Calc. for C_20_H_22_ClCoN_8_S_2_ : C, 45.07; H, 4.16; Cl, 6.65; Co, 11.06; N, 21.02; S, 12.03; Found:  C, 45.25; H, 4.27; Cl, 6.52; Co, 11.21; N, 20.91; S, 11.95. Main IR peaks (cm^−1^) : *ν* (NH) 3122, *ν*(C = C allyl) 1642, *ν*(C = N^2^) 1603, *ν*(C = N^1^) 1578, *ν*(С = N_pyr_) 1556, *ν*(C–S) 776. *μ*_eff_ (292 ± 1K): 0 *μ*_B_; *χ* (CH_3_OH): 78 Ω^−1^ cm^2^ mol^−1^.

### 2.4. Physical Measurements

The chemical elemental analysis, melting points of the thiosemicarbazones, molar conductivity values, and magnetic susceptibilities were determined by specific methods [40]. IR spectra of the studied organic and complexes were recorded on a Bruker ALPHA FTIR spectrophotometer. UV-Vis spectra were measured on Agilent Cary 300 UV-Vis spectrophotometer in 0.1 mM DMSO solutions. The NMR (^1^H and ^13^C) spectra of the studied thiosemicarbazones (*HL*, *HL*^a^, *HL*^b^) were recorded on a Bruker DRX-400 spectrometer (Billerica, MA, USA) DMSO-d_6_ and acetone-d_6_ solutions.

EPR data were recorded using an Elexsys E 500 Bruker spectrometer, operating at a microwave frequency of approximately 9.47 GHz. Spectra were recorded using a microwave power of 10 mW across a sweep width of 200 mT (centered at 320 mT) with modulation amplitude of 0.4 mT. Experiments were carried out at 110 K using a liquid nitrogen cryostat. About 10 *μ*mol of the corresponding compound were dissolved in 1 mL of DMSO to obtain a 10-mM stock solution. The 0.1, 0.2, and 0.5 mM solutions were prepared by dilution of the corresponding stock solution with DMSO. For low concentrations, several scans (4 to 6) were averaged.

### 2.5. X-Ray Crystallography

The single crystal X-ray analysis of three thiosemicarbazones (*HL*, *HL*^a^ and *HL*^b^) and complexes **4** and **5** were carried out on a Xcalibur E CCD diffractometer equipped with a CCD area detector and a graphite monochromator, MoK*α* radiation, at room temperature. Data collection and reduction, and unit cell determination were done by CrysAlis PRO CCD (Oxford Diffraction); the SHELXS97 and SHELXL2014 program packages [41, 42] were used to solve and refine the structures. The non-hydrogen atoms were treated anisotropically (full-matrix least squares method on *F*^2^). The hydrogen atoms were placed in calculated positions and were treated using riding model approximations with Uiso(H) = 1.2Ueq(C), while the oxygen bounded H-atoms were found from differential Fourier maps at an intermediate stage of the structure refinement. These hydrogen atoms were refined with the isotropic displacement parameter Uiso(H) = 1.5Ueq(O). The X-ray data and the details of the refinement of *HL*, *HL*^a^, *HL*^b^, **4** and **5** are summarized in Table 1, and the selected bond lengths are given in Table 2. The selected bond lengths for *HL*^a^, *HL*^b^, and hydrogen bond parameters are given in Tables S1, S2. The geometric parameters were calculated by PLATON program [43] and Mercury software [44] was used for the visualization of structures. The hydrogen atoms that were not involved in the hydrogen bonding were omitted from the generation of the packing diagrams.

### 2.6. Biological Studies

The antiproliferative activity of the studied substances was determined toward a series of cancer cell lines (HL-60 (ATCC CCL-240), BxPC-3 (ATCC CRL-1687), HeLa (ATCC CCL-2), RD (ATCC CCL-136)), and a normal cell line (MDCK (ATCC CCL-34)). These cell lines were cultured in accordance with the previously described method [45]. Determination of the number of viable cells after cultivation in the presence of various concentrations of the studied substances was carried out by the MTS and Resazurin assays according to the previously described method [45]. The 10-mM stock solutions of the studied compounds as well as the reference compound (doxorubicin) were prepared by dissolving 10 *μ*mol of the corresponding compound in 1 mL of DMSO. The 0.1, 1, 10, and 100 *μ*M solutions were prepared by dilution of the stock solutions with the corresponding medium and used for the study of antiproliferative activity.

Antibacterial and antifungal properties of the studied substances were determined on a series of standard strains of Gram-positive microorganisms (*Staphylococcus aureus* (ATCC 25923)), Gram-negative microorganisms (*Escherichia coli* (ATCC 25922)), and fungi (*Candida albicans* (ATCC 10231)) using the method of serial dilutions in liquid broth. Stock solutions of the studied compounds as well as the reference compounds (furacillinum and nystatin) were prepared by dissolving 10 mg of the corresponding compound in 1 mL of DMSO. After that, the starting solutions (1 mg/mL) were prepared by tenfold dilution of the stock solution with nutrition medium (2% of peptonate bullion). The next dilutions were prepared by serial double dilution with the nutrition medium.

The ABTS^•+^ method [46] was applied for the determination of antiradical activity of the studied substances. Standard solutions of ABTS^•+^ radical cations and tested substances as well as the conditions of spectrophotometric measurements and calculations of inhibition were made as described [6]. The 10-mM stock solutions of the studied compounds as well as the reference compound (trolox) were prepared by dissolving 10 *μ*mol of the corresponding compound in 1 mL of DMSO. The 1, 10, 100, and 1000 *μ*M solutions were prepared by the dilution of stock solutions with DMSO. After that, 20 *μ*L of each tested compound solutions was transferred in a 96-well microtiter plate, and 180 *μ*L of ABTS^•+^ working solution was added to receive solutions with final concentrations of tested compounds 0.1, 1, 10, and 100, respectively.

#### 2.6.1. Acute Toxicity Assay against *Daphnia magna*

The general toxicity of the tested compounds was evaluated using *Daphnia magna* (Straus, 1820)*. Daphnia magna* was originated from a culture maintained parthenogenetically at the Institute of Zoology (Laboratory of Systematics and Molecular Phylogeny).

The test design was based on ISO 6341 : 2012. The test-organisms *Daphnia magna Straus* were fed with *Chlorella vulgaris*. These unicellular algae were grown using aseptic technology to exclude contamination of the culture by bacteria, algae or protozoa. *Chlorella vulgaris* were cultivated in Prat's growth medium containing KNO_3_ (1 *μ*M), MgSO_4_·7H_2_O (40 *μ*M), K_2_HPO_4_·3H_2_O (400 *μ*M), FeCl_3_·6H_2_O (3.6 *μ*M) in H_2_O distilled (adjusted the pH to 7.0, autoclaved and stored at 5°C).

*D. magna* was maintained in aerated aqueous straw infusion growth media supplemented with CaCl_2_ (11.76 g/L), NaHCO_3_ (2.59 g/L), KCl (0.23 g/L), and MgSO_4_, 7H_2_O (4.93 g/L). (pH∼7.5 ± 0.2; O_2_ ≥ 6.0 mg/L).

Juveniles were selected according to their size and kept in fresh medium for 24 h. *D. magna* was cultured in Costar® 24-well culture clear sterile multiple well plates covered by a lid to prevent the possibility of contamination and evaporation but at the same time to allow gaseous exchange between air and culture medium. Each well contained 10 daphnids in 1000 *μ*L final volume of each dilution of the tested compounds.

The bioassay was then repeated at the concentrations ranging from 0.1 to 100 *μ*M (0.1, 1, 10, and 100 *μ*M) in order to determine LC_50_ for each compound, including doxorubicin (the positive control). Aqueous straw infusion growth media was used to dilute the stock solutions to the required concentrations. The final test solutions contained up to 0.1% DMSO and had a final volume of 1 mL. A 0.1% solution of DMSO in aerated medium (pH∼7.5 ± 0.2; O_2_ ≥ 6.0 mg/L) was used as a negative control. Throughout the experiment, the juvenile daphnids were incubated at 22 ± 2^0^C, using a 16 h/8 h light/dark cycle (500–1000 lx). The mobility (viability) of the test organisms was observed after the 24-h exposure. The experiment was performed in triplicate.

The daphnids were considered immobilized only if they did not swim during the 15 s which follow gentle agitation of the test and control solutions, even if they could still move their antennae. The percentage of viability (V (%)) of *Daphnia magna* was calculated according to the formula:

(1) $$\begin{array}{c} V\left(\%\right)=\frac{N_{\left(\text{sample}\right)}}{N_{\left(\text{control}\right)}}\times 100. \end{array}$$

N - Number of viability of *Daphnia magna*.

The compounds median lethal concentration that kills 50% of the juvenile daphnids (LC_50_) values were calculated from the dose-response equation determined by the least squares fit method.

## 3. Results and Discussion

The thiosemicarbazone *HL* has been previously described by Zeglis B.M. et al. [39] and has been obtained using the described method. Its yield is 89%. It was characterized by FT-IR, UV–Vis, ^1^H NMR, and ^13^C NMR spectroscopy. Also, its structure as well as structures of other two thiosemicarbazones with different position of pyridine nitrogen atom (*HL*^a^ and *HL*^b^) has been determined using X-ray diffraction analysis. Complexes **1**–**6** were synthesized by the interaction of ethanolic solution of 2-formylpyridine *N*^4^-allylthiosemicarbazone (*HL*) and zinc chloride (**1**), copper(II) salts (**2**–**4**) in a 1 : 1 molar ratio or nickel(II) (**5**) and cobalt(II) (**6**) salts in a 2 : 1 molar ratio. The composition of the thiosemicarbazone *HL* and complexes **1**–**6** has been confirmed using elemental analyses data.

### 3.1. Study of Substances in Solid State

#### 3.1.1. Structural Characterization of Thiosemicarbazones (*HL*, *HL*^a^, *HL*^b^) and Complexes **4** and **5**

**Figure 1:**
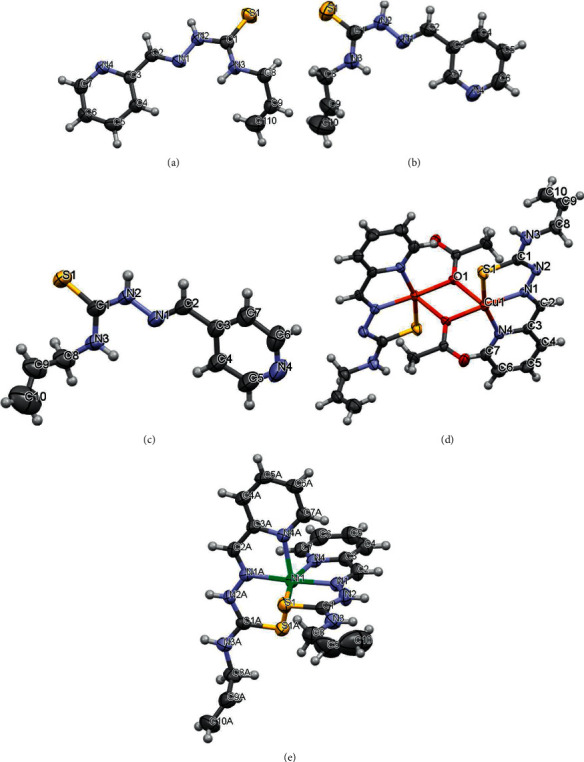
View of (a) *HL*, (b) *HL*^a^, (c) *HL*^b^, (d) **4**, and (e) **5** compounds with atom numbering. Thermal ellipsoids are drawn at 50% probability level.

**Figure 2:**
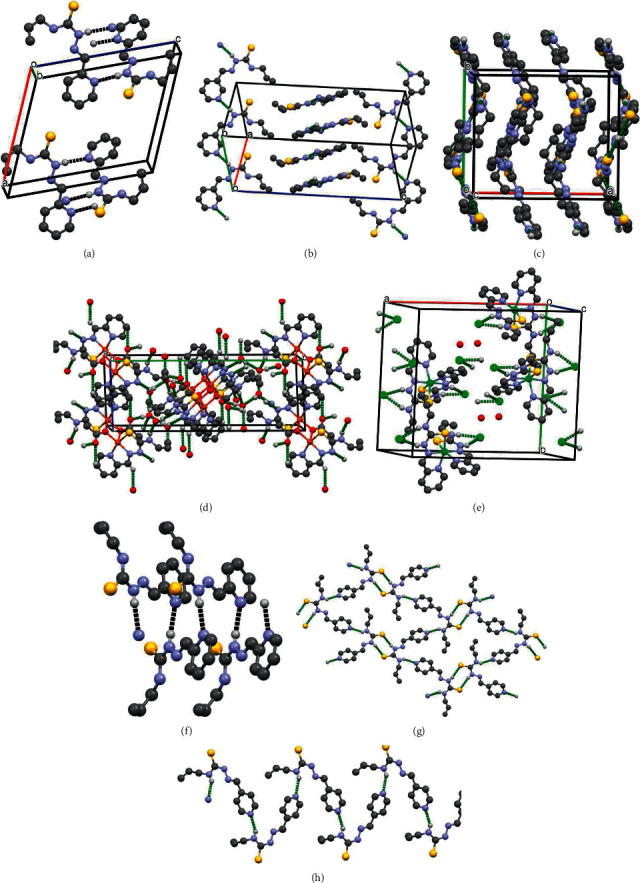
The crystal packing fragments of (a) *HL* (b) *HL*^a^, (c) *HL*^b^, (d) **4**, (e) **5**, (f) chains in *HL*, (g) layers in *HL*^b^, and (h) chains in *HL*^b^.

The X-ray structures of studied thiosemicarbazones and compounds **4** and **5** are presented in Figures 1 and 2. The conformations of *HL*, *HL*^a^, and *HL*^b^, which differ only by position of nitrogen atom N(4) in pyridine ring, are nearly the same. In these molecules, the substituents at the N(2)–C(1) bond are in the Е configuration. The *A*(S(1)-N(1)-N(2)-N(3)-C(1)-C(2)) core is practically planar within 0.06 Å in these ligands and the dihedral angle between given core and pyridine ring ranges from 6.5 to 10. Meanwhile, the *HL*, *HL*^a^, and *HL*^b^ are nonplanar because the C_3_H_5_ substituent in the thiosemicarbazone moiety, the dihedral angles between the mean planes of *A*, and (С(8)–С(10)) fragments lie in interval 46.4 ÷ 52.0°.

The “shifting” of nitrogen atom N(4) in the pyridine rings has led to different hydrogen bonds (HB) architecture in the crystal structures of *HL*, *HL*^a^, and *HL*^b^. In the crystal, the ligand *HL* forms the zigzag chains along *b*-axis, where the molecules are linked by N(2)-H…N(4) hydrogen bonds, while in *HL*^a^ the similar HB forms two types of chains, which are oriented along different diagonals of (110) plane (Table S2, Figures 2(a), 2(b) and 2(f)). In *HL*^b^, there are three independent molecules (**L1**, **L2**, **L3**) in the asymmetric unit cell. **L2** forms layers parallel to (011) plane due to N2A-H…S1A and N3A-H…N4 hydrogen bonds, while **L1** and **L3** are joined into the chains along *b*-axis through the N3-H⋯ N4B and N3B-H⋯ N4 HB. The chains and layers are further alternated along *a-*axis (Table S2, Figures 2(c), 2(g) and 2(h)).

In complexes **4** and **5,** the ligand *HL* acts as mononegative and neutral tridentate around the metallic ions, respectively, through an SNN set of donor atoms. Deprotonation of N2 atom in **4** has led to the decrease of N2-C1 bond distance if compared with that in *HL*. The bond lengths S1-C1 in **4** and **5** are increased due to the coordination of suphur atoms to central metals and the maximal changing of this bond is observed in **4** which is equal to 1.734(5) (Table 2). However, the composition of the coordination polyhedron of the central atom in these compounds is different. In the crystal, the copper complexes form the centrosymmetric dimers where the monomers are hold together by bridge acetate molecules. The central atom in **4** is five-coordinated in a distorted square–pyramidal coordination geometry. Its basal plane includes three donor atoms of the L and oxygen atom O(1) of coordinated acetate molecule (Figure 2(d), Table 2). The deviations of these atoms from their mean plane are within 0.017 Å, while the Cu atom deviates from this plane by 0.1 Å toward the apex of pyramid. This apex of the metal's coordination pyramid in **4** is occupied by oxygen atom O(1) of adjacent acetate molecule with a distance of 2.429(3) Å. The polyhedral volume is related to the distortion of coordination polyhedral and its value for the copper complex is 6.765 Å^3^. The structural characterization of **5** revealed that the nickel atom in this complex is in a distorted octahedral environment, being surrounded by two *HL* ligands (Figure 1(e), Table 2). The octahedral volume of Ni complex is equal to 13.338 Å^3^. In the crystal of **4,** there are water molecules in the outer coordination sphere which are joined by HB. In turn, these water molecules bound the neutral centrosymmetric dimers forming the 3D hydrogen bonding networks where dimers are linked also by C2-H⋯ O2 HB (Table S2, Figure 2(d)). In the crystal of **5,** the outer sphere contains both water molecules and negative chlorine ions. These chlorine ions form the hydrogen bonds with positively charged octahedral nickel complexes (Table S2, Figure 2(e)) *via* bifurcated N2-H⋯ Cl and N3-H⋯ Cl bonds.

#### 3.1.2. Infrared Spectra

The process of formation of complexes **1**–**6** leads to the appearance of a number of changes in the IR spectra in comparison with the IR spectrum of free ligand *HL*. A change in the position of the *ν*(*С* = N_pyr_), *ν*(C = N^1^) bands in the IR spectra of **1**–**6** as well as the disappearance of the *ν*(*С* = *S*) band in the spectra of **1**–**4** and **6** is observed. In case of complex **5,** the *ν*(*С* = *S*) does not disappear, but is only shifted by 49 cm^−1^. The *ν*(*С* = N_pyr_) band is shifted by 17–45 cm^−1^ toward lower wavenumbers comparing with the free ligand *HL* (1583 cm^−1^). The *ν*(C = N^1^) band is shifted by 14–39 cm^−1^. One *ν*(NH) band disappears in the IR spectra of complexes **1**–**4** and **6**. All the above changes indicate [47] that the azomethine and pyridine nitrogen atoms as well as the sulphur atom of the thiosemicarbazone *HL* are involved in the coordination process. While in the complexes **1**–**4** and **6,** the thiosemicarbazone is in the thiol deprotonated form and in the composition of complex **5** thiosemicarbazone remains in the non-deprotonated thione form.

#### 3.1.3. Magnetic Study

A magnetochemical study was carried out in order to determine the presence of interaction between copper atoms in complexes **2**–**4**. While the effective moment of complex **4** (1.25 *μ*_B_) is lower than the spin only value (1.73 *μ*_B_), which indicates the presence of interaction between copper atoms that is consistent with the determined structure by X-ray diffraction analysis, the effective magnetic moments of complexes **2** and **3** exceed the spin only value (1.85 *μ*_B_ and 1.81 *μ*_B_, respectively). These values indicate that there is no interaction between copper atoms in complexes **2** and **3**, so that they should possess a monomeric structure [48].

The magnetic moment of complex **6** was measured in order to determine the oxidation state of cobalt. The complex **6** turned out to be diamagnetic, which is possible only if cobalt atom is in the +3 oxidation state in an octahedral ligand environment [49]. So, cobalt(II) undergoes oxidation by atmospheric oxygen during the synthesis process.

The magnetic moment value of nickel complex **5** (2.84 *μ*_B_) corresponds to two unpaired electrons and octahedral ligand environment that fully corresponds to the structure of the given substance determined by X-ray diffraction analysis.

### 3.2. Study of Substances in Solution

#### 3.2.1. Molar Electrical Conductivity

Molar conductivity values have been measured for 1 mM solutions of the corresponding complexes. Molar conductivity values of **1**–**4** and **6** are in the range 32–78 Ω^−1^ cm^2^ mol^−1^ which indicates that they are 1 : 1 electrolytes [50]. It means that in case of complexes **1**–**4**, the corresponding anions (Cl^−^ (**1**, **2**), Br^−^ (**3**), CH_3_COO^−^ (**4**)) are substituted with solvent molecules during dissolution process. The obtained values for copper(II) complexes in methanol solution are rather low but rapidly change to normal values for 1 : 1 electrolytes upon adding of DMSO in the methanol solution. It indicates that methanol does not cause a complete substitution of anions from the inner sphere with the solvent molecule, while DMSO molecules shift this equilibrium toward the removal of anions from the inner sphere. Nickel complex **5** represents 1 : 2 electrolytes (*χ* = 158 Ω^−1^ cm^2^ mol^−1^).

#### 3.2.2. UV-Vis Spectra

UV-Vis spectrum of 0.1 mM DMSO solution of *HL* (Figure S7) contains a maximum at 328 nm. The corresponding spectrum of zinc(II) complex (**1**) contains two maxima at 325 and 397 nm. The spectra of complexes **2**–**4** contain two maxima at 300 and 409 nm and a shoulder at ca 335 nm. The corresponding spectrum of nickel(II) complex contains two maxima at 320 and 419 nm, while the spectrum of cobalt(III) complex contains two maxima at 310 and 371 nm and one shoulder at 416 nm. Thus, by comparing the results of the UV-Vis spectra of the complexes and the initial thiosemicarbazone in the 0.1 mM DMSO solutions, it can be concluded that thiosemicarbazpone moiety remains coordinated to the corresponding 3*d* metal ions when dissolved in DMSO [51]. It is interesting that the copper complexes have almost identical spectra. This may indicate that in the process of dissolution in DMSO the anion of the acid residue regardless of its nature is replaced by a DMSO molecule and a complex cation containing a copper atom and an L^−^ ligand is formed [52]. This is consistent with the change in the electrical conductivity of methanol solutions of copper complexes that occur upon the addition of DMSO.

#### 3.2.3. EPR Study

**Figure 3:**
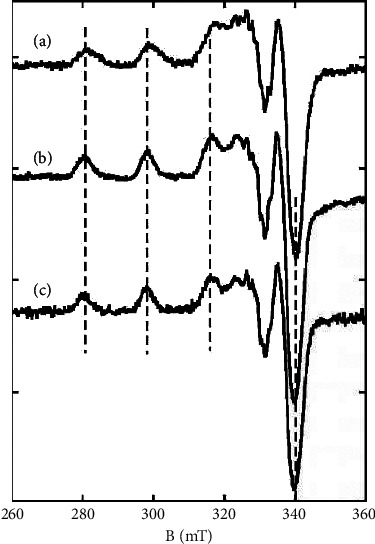
9.5-GHz EPR spectra of complexes **2** (a), **3** (b), and **4** (c). Dotted lines indicate the position of the hyperfine lines.

The EPR signatures of complexes **2**–**4** were recorded in a 9-GHz band and the spectra obtained at 0.2 mM in DMSO are shown in Figure 3. They are all very similar with a classical pattern for Cu(II) complexes lying in a square-based environment. The g//factor equals 2.20, while the hyperfine splitting equals 185 ± 5 10^−4^ cm^−1^ (dotted lines). The parameters are in line with a equatorial donor set of [2N,S,O] [53] and strongly reminiscent with those of a Cu(II) complex bound to three nitrogen atoms and one thiolate ligand [54]. In the perpendicular region, super-hyperfine lines are observed that mirror the interaction of the Cu(II) center with nitrogen atoms form the ligand. While the spectra of **3** and **4** are virtually superimposable and independent of the recording concentration, that of **2** shows broader hyperfine lines that witness the presence of a second minor species, the proportion of which increases with the concentration (Figure S8). These observations indicate, in line with the UV-Vis data, that the exogenous ligand is freed in solution and replaced by a solvent molecule with the exception of Cl^−^ in **2** that partly stays bound.

### 3.3. Biological Activity

#### 3.3.1. Antiproliferative Activity and *In Vivo* Toxicity

The antiproliferative activity of thiosemicarbazones *HL*, *HL*^a^, and *HL*^b^ has been tested toward human leukemia HL-60 cell line. As it can be seen from Table 3, the position of nitrogen atom in the pyridine moiety of studied thiosemicarbazones has a tremendous influence on their antiproliferative activity. While *HL* inhibits the growth and proliferation of HL-60 cells by ca 100% at 10 and 1 *μ*M concentrations, and *HL*^a^ and *HL*^b^ practically do not affect their proliferation. Because changing of the position of nitrogen atom in pyridine moiety leads to a complete disappearance of antiproliferative properties, only 2-formylpyridine *N*^4^-allylthiosemicarbazone was used for the synthesis of complexes. Subsequently, antiproliferative activity of the thiosemicarbazone *HL* and complexes **1**–**6** has been tested toward four cancer cell lines (human cervical epithelial (HeLa), human muscle rhabdomyosarcoma spindle and large multinucleated (RD), human leukemia (HL-60),, human epithelial pancreatic adenocarcinoma (BxPC-3)), and one normal cell line (normal kidney epithelial (MDCK)) (Table 4).

The nature of the central atom has a major influence on the antiproliferative activity of the corresponding complex. The activity of zinc(II) complex (**1**) toward HL-60 cells is practically equal to the activity of the free ligand *HL*. Meanwhile, the activity of cobalt complex (**6**) is significantly lower, but copper (**2**–**4**) and nickel (**5**) complexes are much more active compared to the activity of *HL*. Complexes **2**–**5** inhibit the proliferation of HL-60 cells by ca 100% at 10 and 1 *μ*M concentration and also manifest inhibitory activity (24–55%) at 0.1 *μ*M concentration.

It is known from the literature sources that copper(II) complexes in many cases manifest the best antiproliferative activity. In this series of complexes, the nickel complex **5** showed the highest inhibitory activity toward HL-60 cells.

The copper complexes **2**–**4** manifest the highest activity toward the studied series of cancer cell lines. Moreover, these complexes are superior in antiproliferative activity to doxorubicin that is used in medical practice. Also, zinc complex **1** manifests high activity toward RD cells which is approximately equal to the activity of the most active copper complex **4**.

Antiproliferative activity of these compounds toward normal MDCK cell line has been studied in order to determine the selectivity of their anticancer activity. Nickel (**5**) and cobalt (**6**) complexes practically do not affect the growth and proliferation of this normal cell line. Meanwhile, complex **6** does not possess promising anticancer activity, and nickel complex **5** manifests the best activity toward human leukemia cells and its activity is highly selective with selectivity index (SI) more than 1000. Also, free ligand *HL* and zinc complex **1** have high values of SI toward HL-60 that significantly exceed the selectivity of doxorubicin used in medical practice.

As the obtained results of anticancer activity of the studied series of complexes and their selectivity are of both theoretical and practical interest, their toxicity has been studied *in vivo* on *Daphnia magna* (Table 5). The percentage of viability has been measured after 24 h of staying in the environment with different concentrations of studied substances. As it can be seen from Table 5, the nickel complex (LC_50_ > 100 *μ*M) practically does not affect *Daphnia magna*, while copper and zinc complexes as well as *HL* are toxic at 10 and 100 *μ*M concentrations and their LC_50_ values are in the range of 1.0–3.5 *μ*M.

#### 3.3.2. Antibacterial and Antifungal Activity

Despite the fact that the role of the virus and bacteria in the development of cancer is underestimated, antimicrobial prophylaxis during cancer therapy is important for reducing some side effects and the risk of death due to infection [55, 56].

It is known from the literature that thiosemicarbazones and biometal complexes with these ligands in many cases also exhibit antimicrobial activity. Therefore, the antimicrobial and antifungal properties of the synthesized compounds have been studied. Three strains have been selected for this study: *S. aureus* (Gram-positive bacterial strain), *E. coli* (Gram-negative bacterial strain), and *C. albicans* (fungal strain).

The obtained results are presented in Table 6 in the form of minimum inhibitory, bactericidal, and fungicidal concentrations. Table 6 also shows data on nystatin and furacillinum [11] to compare the activity of the synthesized compounds.

As it can be seen from the obtained data, zinc and copper complexes manifest the highest antimicrobial activity. While their activity toward Gram-positive bacterial strain is only 2–4 times higher than the activity of thiosemicarbazone *HL*, the activity toward Gram-negative bacterial strain already exceeds 4–16 times the corresponding activity of free ligand. Copper complexes **2**–**4** also manifest promising antifungal activity that exceeds the activity of nystatin.

The nickel (**5**) and cobalt (**6**) complexes manifest much lower antimicrobial and antifungal activities comparing with free ligand *HL*, complexes **1**–**4**, and standard substances.

#### 3.3.3. Antiradical Activity

Free radicals are involved in many harmful biological processes, such as protein denaturation and lipid peroxidation, in the pathogenesis of various human diseases [57]. Therefore, it is of interest to study the antiradical activity of the synthesized substances in order to find out if they can reduce the concentration of free radical and protect human body from the oxidative stress. A standard ABTS^•+^ method has been used to determine these properties (Table 7).

The thiosemicarbazones *HL*, *HL*^a^, and *HL*^b^ manifest high antiradical activity toward ABTS^•+^ with IC_50_ values 14.2–20.6 *μ*M comparing to the standard antioxidant trolox (IC_50_ value 33 *μ*M [58]). Meanwhile complexes of copper (**2**–**4**) and cobalt (**6**) manifest much lower antiradical activity (IC_50_ > 40 *μ*M). The nickel(II) complex (**5**) manifests the highest antiradical activity with IC_50_ value 8.6 ± 0.1 *μ*M.

### 3.4. Electronic Properties of Studied Compounds

The electronic structures and maps of molecular electrostatic potential (MEP) of *HL*, *HL*^a^, *HL*^b^, **4**, **5** compounds have been calculated by Density Functional Theory (DFT) of Gaussian 16 suite of quantum chemical codes based on *B3LYP* level of theory [59]. The 6-31G and LANL2DZ basis sets were used for ligands and metal atoms, respectively. The spin-polarized calculations were performed for compound **4**. The molecular electrostatic potential was generated through a constant value of electron density. The values of MEP were calculated with the help of Multiwfn software [60] using the wave functions generated by the Gaussian.

#### 3.4.1. Molecular Electrostatic Potential

**Figure 4:**
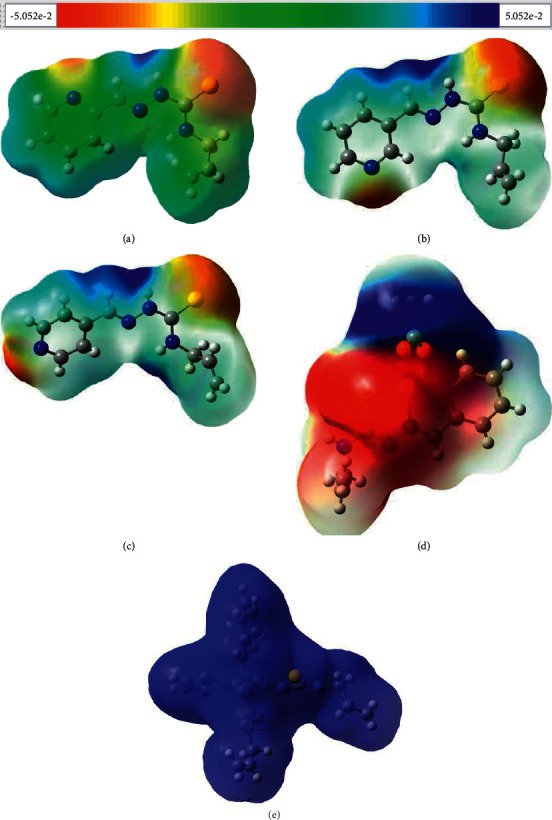
Electrostatic potentials mapped on the molecular surfaces of (a) *HL*, (b) *HL*^a^, (c) *HL*^b^, (d) **4**, and (e) **5** . The values of MEPs range from −31.7 to 31.7 kcal/mol.

The molecular electrostatic potential is used to study and predict the molecular reactive centers. MEP is related to the electron density, which is descriptor for identifying nucleophilic and electrophilic attack sites as well as intermolecular hydrogen bonding [61]. The electrostatic potential is also applied to analyze the processes based on the ‘‘recognition” of one molecule by another, as in drug-receptor, and enzyme–substrate interactions [62, 63]. The molecular electrostatic potential surfaces of all studied molecules were mapped, using crystallographically determined geometries, and presented in Figure 4 The MEPs increase in the order −31.7 kcalˑmol^−1^ = red < yellow < green < blue = 31.7 kcalˑmol^−1^. The negative (red and yellow) regions of MEP are related to electrophilic reactivity and the positive (blue) regions to nucleophilic reactivity. In *HL*, *HL*^a^, and *HL*^b^, the negative electrostatic potential is located at the regions of nitrogen atoms of pyridine rings and in the vicinity of sulphur atoms, while the positive MEP covers the outer tips of the CH- and NH-groups in the thiosemicarbazone moieties. However, the values of MEP of N4 depend on its position in the pyridine rings, and the negative charges of electrostatic potentials are increased under “shifting” of N4 in these ligands as follows: −33.744, 39.758 and −40.674 kcal/mol. The values of S1 atoms MEPs are equal to −33.021, −32.186, and −29.702 kcal/mol, respectively. Thus, such distribution of negative MEPs may affect halogen bonding architecture of *HL*, *HL*^a^, and *HL*^b^ as described in Section 3.1.1. In compound **4,** the negative MEP covers the metallacycle (Cu1S1N1N2C1) and oxygen atoms of acetate molecule, and the positive region is over the methyl group. In compound **5,** it is observed only the strong positive electrostatic potential surrounding over whole molecule due to the positive charge of this complex.

#### 3.4.2. Analysis of Frontier Orbitals

**Figure 5:**
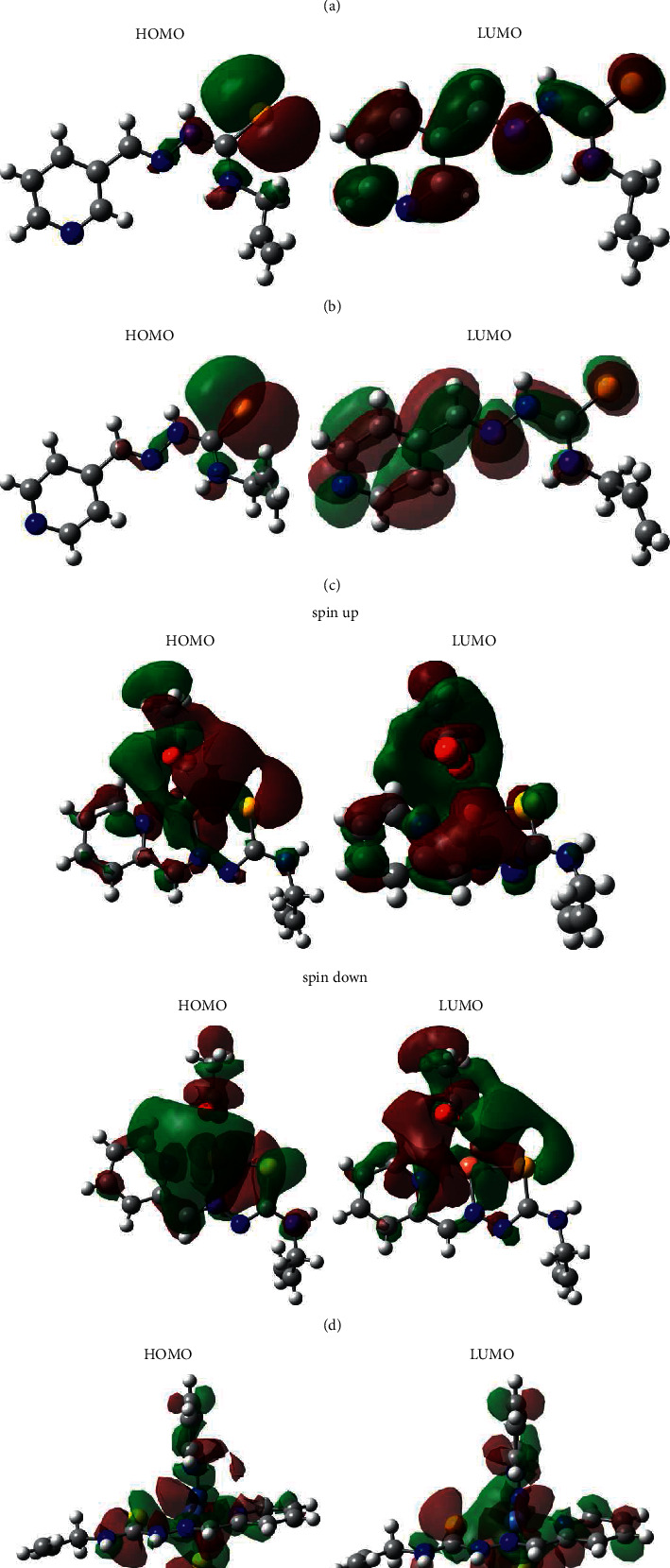
View of frontier molecular orbitals (HOMO and LUMO) for (a) *HL*, (b) *HL*^a^, (c) *HL*^b^, (d) **4**, and (e) **5**.

It is known that the structure–activity relationship models for understanding the biological activity and toxicity of molecules may be generated in terms of global and local reactivity descriptors within a conceptual density functional theory framework [64]. The global reactivity indexes of studied compounds were estimated based on the one-electron energies of the frontier molecular orbitals (FMO) [65, 66]. The calculated molecular descriptors and the relationships between them for studied compounds as well as biological activities are summarized in Table 8. The high occupied molecular orbitals (HOMO) and lower unoccupied molecular orbitals (LUMO) are referred to as frontier molecular orbital and act as an electron donor and acceptor, respectively. The energy gap between the HOMO and LUMO is related to kinetic stability as well as the chemical reactivity of the compound, the molecules having large/small values of energy gap are known as hard/soft molecules, as well as HOMO–LUMO band gap supports the bioactive property of the molecule [67]. The contour plots of the ground state FMO are shown in Figure 5. The HOMOs of *HL*, *HL*^a^, and *HL*^b^ are located mainly on the sulphur atoms with a small contribution of nitrogen atoms of the chains, while the LUMOs are distributed in thiosemicarbazone moieties and pyridine rings. In compound **4,** the spin np HOMO is localized on the central atom and acetate molecule, and the spin down HOMO is located mainly on metallocycles with some contribution of oxygen atoms. The spin np LUMO is distributed on acetate molecule, metallocycles, and pyridine ring, whereas the spin down LUMO is located mainly on acetate molecule and copper atom. It is interesting to observe that both HOMO and LUMO of compound **5** are substantially distributed over the conjugation planes of compound **5** which include metallocycles and pyridine rings. Compound **5** has the lowest energetic gap which allows it to be the softest molecule among the studied ones. Compounds **4** and **5** with high-lying E_HOMO_ and low-lying E_LUMO_ are the best electron donor and the electron acceptor, respectively, in a given series of compounds. The absolute electronegativity (*χ*) and the absolute hardness (*η*) are related to the one-electron orbital energies of the HOMO and LUMO. It is known that the stability of molecules is related to hardness and electronegativity is the power of an atom in a molecule to attract electrons to itself. The chemical hardness value of compound **5** is lesser among all the molecules, and it is found to be more reactive than all the studied compounds. This compound possesses higher electronegativity value than all compounds, so it is the best electron acceptor. The electrophilicity reactivity index (*ɷ*) measures the stabilization in energy when the system becomes saturated by electrons coming from environment. A good electrophile is characterized by a high value of *µ* (chemical potential) and a low value of *η* (chemical hardness). The higher electrophilicity of **5** is displaying that this compound would be more susceptible for nucleophilic attack because the electrophilicity is generally proportional to electron-poorness. The analyses of dependencies between the global reactivity indexes of studied compounds and their biological activities revealed that the experimental data were related to the theoretical ones (Table 8). It was found that the energetic gaps and hardness correlate with antiproliferative activities. The values of ionization potential, electron affinity, electronegativity, and electrophilicity are related to antibacterial and antifungal activities. The energies of LUMO and HOMO as well as chemical potentials correlate with antiradical activity. However, the most significant values of molecular descriptors and corresponding correlations with biological activities were observed for compound **5**.

## 4. Conclusion

Three thiosemicarbazones (2-, 3-, and 4-formylpyridine *N*^4^-allylthiosemicarbazones) and six complexes of Zn(II), Cu(II), Ni(II), and Co(III) have been prepared and characterized using different chemical, physical, and physico-chemical methods. The structures of *HL*, *HL*^a^, *HL*^b^ and complexes **4** and **5** have been determined using single-crystal X-ray diffraction analysis. The *HL* ligand remains in a non-deprotonated form in the composition of nickel(II) complex and deprotonates in the case of compounds **1**–**4** and **6**. It was found that the position of nitrogen atom N(4) in the pyridine rings affects the different hydrogen bonds architecture in the crystal structures of *HL*, *HL*^a^, and *HL*^b^. The copper atom in **4** is five-coordinated in a distorted square–pyramidal coordination geometry and in the crystal this compound forms the centrosymmetric dimers where the monomers are hold together by bridge acetate molecules. In **5,** the nickel atom is in a distorted octahedral environment being surrounded by two *HL* ligands.

The anticancer activity of the synthesized zinc, copper, and nickel compounds (**1**–**5**) is of interest. These complexes show high activity against HL-60, HeLa, BxPC-3, and RD cancer cell lines. Nickel complex **5** manifests high antiproliferative activity toward HL-60 cells and practically does not affect normal cells showing the highest selectivity toward this cell line exceeding the selectivity of doxorubicin used in medical practice, and also the activity of other substances of thiosemicarbazone class such as triapine [68] that undergoes preclinical and clinical testing as anticancer drug. It also shows the lowest *in vivo* toxicity. It makes substance **5** promising for preclinical and clinical trials as anticancer substance. The copper and zinc complexes (**1**–**4**) also manifest high selective activity toward cancer cells toward a wide range of studied cancer cells, but they have a higher inhibitory activity toward normal cell line and are more toxic.

In addition to high anticancer properties, zinc and copper complexes also exhibit high antimicrobial properties with MIC/MBC/MFC values in the range of 0.12–250 *μ*g/mL).

The molecular electrostatic potential and the global reactivity indexes were calculated at B3LYP level of theory and then discussed in terms of their biological activities. It was found that the different reactivity descriptors of studied compounds strongly correlate with their biological activity and such dependences provide information for structure-activity study to design the biologically important molecules that can be applied in the pharmacy and medicine.

## Figures and Tables

**Table 1:** Crystal and structure refinement data for *HL*, *HL*^a^, *HL*^b^, **4**, and **5**.

Compound	*HL*	*HL* ^a^	*HL* ^b^	**4**	**5**
CCDC	2129913	2129912	2129910	2129911	2129914
Empirical formula	C_10_H_12_N_4_S	C_10_H_12_N_4_S	C_10_H_12_N_4_S	C_24_H_36_Cu_2_N_8_O_8_S_2_	C_20_H_26_Cl_2_N_8_NiOS_2_
Formula weight	220.3	220.3	220.3	755.83	588.2
Temperature/K	293	293	293	293	293
Radiation, 0.71073 (Å)	MoK*α*	MoK*α*	MoK*α*	MoK*α*	MoK*α*
Crystal system	Monoclinic	Monoclinic	Monoclinic	Monoclinic	Monoclinic
Space group	P2_1_	C2/c	P2_1_/c	P2_1_/c	P2_1_/c
a (Å)	10.919 (2)	10.121 (2)	12.5635 (6)	8.6275 (13)	19.3092 (8)
b (Å)	4.2978 (7)	10.003 (2)	10.6121 (7)	22.8273 (17)	16.5371 (7)
c (Å)	12.8929 (16)	22.991 (3)	26.1172 (8)	8.9748 (9)	8.4168 (4)
*α* (°)	90	90	90	90	90
*β* (°)	112.089 (17)	91.801 (16)	93.871 (3)	115.776 (15)	97.227 (5)
*γ* (°)	90	90	90	90	90
*V* (Å^3^)	560.63 (17)	2326.5 (7)	3474.1 (3)	1591.7 (4)	2666.3 (2)
*Z*	2	8	12	2	4
*ρ*_calc_ (g cm^−3^)	1.305	1.258	1.264	1.577	1.465
*μ*Mo (mm^−1^)	0.261	0.252	0.253	1.525	1.114
*F*(000)	232	928	1392	780	1216
Crystal size (mm)	0.3 × 0.35 × .27	0.25 × 0.3 × 0.35	0.25 × 0.3 × 0.35	0.3 × 0.2 × 0.35	0.35 × 0.003 × 0.00
*θ* range (°)	3.2 : 5.0	3.0 : 25.0	3.0 : 25.5	2.9, 25.0	3.0 : 25.5
Indexes ranges, h	−7 : 13	−12 : 6	−14 : 15	−10 : 10	23 : 13
K	−5 : 5	−6 : 11	−12 : 8	−27 : 26	−19 : 19
L	−14 : 15	−24 : 27	−31 : 22	−10 : 10	−10 : 10
Reflections collected	1959	3683	12768	6491	9602
Independent reflections	1570	2050	6422	2819	4947
R(int)	0.027	0.062	0.034	0.045	0.04
Reflections with *I* > 2*σ* (I)	1323	735	3543	1984	3229
Number of refined parameters	136	136	403	214	313
Goodness-of-fit, F^2^ (S)	1.0	0.87	1.02	1.1	1.01
*R1* (for *I* > 2*σ* (I))	0.0479	0.0656	0.0608	0.048	0.0605
*R1w*	0.1110	0.089	0.1328	0.0508	0.1371
R(for all reflections)	0.0581	0.2117	0.1328	0.0812	0.1001
*R1w*	0.1203	0.1236	0.1574	0.0551	0.1577
Δ*ρ*max/Δ*ρ*min (e·Å^−3^)	−0.18, 0.23	−0.15, 0.20	−0.53, 0.59	−0.45, 0.47	−0.45, 0.76

**Table 2:** Selected bond lengths in **4, 5**, (M = Cu1, Ni1), and *HL* (in brackets).

Bond	d, Å		
	**4**	**5**	
M-S1	2.2739 (12)	2.4138 (15)	2.4562 (14)
M-O1	1.954 (2)		
M-N1	1.966 (3)	2.016 (4)	2.007 (4)
M-N4	2.061 (3)	2.089 (4)	2.123 (3)
M-O1a	2.429 (3)		
S1-C1	1.734 (5) (1.673 (5))	1.683 (4)	1.696 (4)
N1-N2	1.364 (4) (1.365 (3))	1.347 (6)	1.357 (5)
N1-C2	1.291 (4) (1.280 (6))	1.281 (6)	1.272 (6)
N2-C1	1.334 (4) (1.365 (6))	1.355 (6)	1.346 (6)
N4-C3	1.361 (4) (1.354(5))	1.351 (7)	1.350 (5)
C2-C3	1.442 (5) (1.465 (7))	1.445 (7)	1.453 (7)

**Table 3:** Antiproliferative activities of some synthesized compounds on human leukemia HL‐60 cell line at three concentrations.

Compound	Inhibition of cell proliferation, (%)			IC_50_, *μ*M
	10 *μ*M	1 *μ*M	0.1 *μ*M	
*HL*	100.3 ± 2.2	99.6 ± 0.4	1.8 ± 0.1	0.30 ± 0.02
*HL* ^a^	6.1 ± 0.3	1.5 ± 0.5	0.9 ± 0.2	—
*HL* ^b^	10.1 ± 0.5	9.4 ± 0.6	2.3 ± 0.4	—
[Zn(H_2_O) (L)Cl] (**1**)	102.4 ± 1.1	101.1 ± 3.7	3.0 ± 0.4	0.20 ± 0.03
[Cu(L)Cl] (**2**)	99.4 ± 0.6	99.2 ± 0.2	40.2 ± 2.8	0.10 ± 0.06
[Cu(L)Br] (**3**)	100.0 ± 0.1	100.0 ± 0.3	24.5 ± 1.9	0.10 ± 0.04
[Cu_2_L_2_(CH_3_COO)_2_]·4H_2_O (**4**)	100.0 ± 0.2	99.8 ± 0.2	32.0 ± 2.3	0.10 ± 0.02
[Ni(*HL*)_2_]Cl_2_·H_2_O (**5**)	108.5 ± 0.5	94.9 ± 0.7	54.5 ± 1.1	0.09 ± 0.01
[Co(L)_2_]Cl (**6**)	24.1 ± 0.6	6.9 ± 1.7	4.1 ± 0.3	>10
Doxorubicin	95.0 ± 0.6	92.9 ± 0.7	16.5 ± 1.8	0.2 ± 0.1

**Table 4:** IC_50_ values of *HL* thiosemicarbazone and complexes **1**–**6** toward MDCK, HL-60, HeLa, BxPC-3, and RD cell lines.

Compound	MDCK	*HL*-60		HeLa		BxPC-3		RD	
	IC_50_, *μ*M	IC_50_, *μ*M	SI^a^	IC_50_, *μ*M	SI^a^	IC_50_, *μ*M	SI^a^	IC_50_, *μ*M	SI^a^
*HL*	>100	0.30 ± 0.02	>300	>100	—	>100	—	1.10 ± 0.03	>90
[Zn(H_2_O) (L)Cl] (**1**)	38.5 ± 4.0	0.20 ± 0.03	193	1.4 ± 0.3	28	10.0 ± 0.3	3.9	0.06 ± 0.01	640
[Cu(L)Cl] (**2**)	3.0 ± 0.4	0.10 ± 0.06	30	0.40 ± 0.03	7.5	0.05 ± 0.02	60	0.13 ± 0.02	23
[Cu(L)Br] (**3**)	6.2 ± 1.4	0.10 ± 0.04	62	0.30 ± 0.03	21	0.06 ± 0.01	103	0.2 ± 0.1	31
[Cu_2_L_2_(CH_3_COO)_2_]·4H_2_O (**4**)	2.0 ± 0.2	0.10 ± 0.02	20	0.20 ± 0.02	10	0.02 ± 0.01	100	0.05 ± 0.01	40
[Ni(*HL*)_2_]Cl_2_·H_2_O (**5**)	>100	0.09 ± 0.01	>1000	>100	—	3.6 ± 0.9	>27	2.9 ± 0.4	>34
[Co(L)_2_]Cl (**6**)	>100	>10	—	2.5 ± 0.5	>40	4.1 ± 0.5	>24	>100	—
Doxorubicin	7.1 ± 0.3	0.2 ± 0.1	35.5	10.0 ± 0.4	0.71	3.7 ± 0.3	1.9	16.2 ± 0.6	0.44

^a^SI–selectivity index.SI=(IC_50_(MDCK)/*IC*_50_(cancer cell line))

**Table 5:** The percentage of viability (V) of *Daphnia magna* in the presence of different concentrations of studied compounds and the corresponding LC_50_ values.

Compound	V, (%)				LC_50_, *μ*M
	100 *μ*M	10 *μ*M	1 *μ*M	0.1 *μ*M	
*HL*	0.0	0.0	56.4 ± 7.3	97 ± 7.3	1.0 ± 0.1
[Zn(H_2_O) (L)Cl] (**1**)	0.0	0.0	56.4 ± 7.3	102.6 ± 0.0	1.0 ± 0.1
[Cu(L)Cl] (**2**)	0.0	0.0	97.4 ± 7.3	102.6 ± 0.0	3.5 ± 2.8
[Cu(L)Br] (**3**)	0.0	0.0	97.4 ± 7.3	102.6 ± 0.0	3.5 ± 2.8
[Cu_2_L_2_(CH_3_COO)_2_]·4H_2_O (**4**)	0.0	0.0	66.7 ± 7.3	76.9 ± 7.3	1.3 ± 0.5
[Ni(*HL*)_2_]Cl_2_·H_2_O (**5**)	87.2 ± 7.3	97.4 ± 7.3	102.6 ± 0.0	102.6 ± 0.0	>100
[Co(L)_2_]Cl (**6**)	35.9 ± 7.3	92.3 ± 0.0	97.4 ± 7.3	97.4 ± 7.3	65.4 ± 11.8
Doxorubicin	0.0	25.6 ± 7.3	76.9 ± 7.3	87.2 ± 7.3	3.3 ± 1.1

**Table 6:** Antibacterial and antifungal activities of *HL* thiosemicarbazone and copper complexes **1**–**6** as MIC^a^/MBC^b^/MFC^c^ values (*μ*g/mL).

Compound	*S. aureus (G+)*		*E. coli (G-)*		*C. albicans*	
	MIC	MBC	MIC	MBC	MIC	MFC
*HL*	0.488	0.977	31.3	62.5	62.5	500
[Zn(H_2_O) (L)Cl] (**1**)	0.244	0.488	3.91	7.81	31.3	250
[Cu(L)Cl] (**2**)	0.122	0.244	3.91	7.81	15.6	31.3
[Cu(L)Br] (**3**)	0.244	0.488	7.81	15.6	7.81	15.6
[Cu_2_L_2_(CH_3_COO)_2_]·4H_2_O (**4**)	0.244	0.488	1.95	3.91	31.3	62.5
[Ni(*HL*)_2_]Cl_2_·H_2_O (**5**)	15.6	31.3	1000	—	500	—
[Co(L)_2_]Cl (**6**)	62.5	125	250	500	62.5	250
Furacillinum	9.3	9.3	18.5	37.5	—	—
Nystatin	—	—	—	—	80	80

*S. aureus* (*Staphylococcus aureus*, ATCC 25923); *E. coli* (*Escherichia coli*, ATCC 25922); *C. albicans* (*Candida* albicans, ATCC 10231). ^a^MIC–minimum inhibitory concentration; ^b^MBC—minimum bactericidal concentration; ^c^MFC, minimum fungicidal concentration. G(-): Gram-negative bacteria; G(+): Gram-positive bacteria.

**Table 7:** IC_50_ values of the synthesized substances toward ABTS^•+^ radical cation.

Compound	IC_50_, *μ*M
*HL*	14.2 ± 1.8
*HL* ^a^	19.1 ± 0.9
*HL* ^b^	20.6 ± 1.1
[Zn(H_2_O) (L)Cl] (**1**)	24.7 ± 1.3
[Cu(L)Cl] (**2**)	>100
[Cu(L)Br] (**3**)	43.0 ± 2.1
[Cu_2_L_2_(CH_3_COO)_2_]·4H_2_O (**4**)	40.3 ± 1.8
[Ni(*HL*)_2_]Cl_2_·H_2_O (**5**)	8.6 ± 0.1
[Co(L)_2_]Cl (**6**)	>100
Trolox	33.0 ± 0.7

**Table 8:** Global chemical reactivity indices (eV), and the biological activities of studied compounds and relationship between them. The biological activities (ID_50_) were taken from Tables 3, 6, and 7. EA–and IP are the electron affinity and ionization potential, respectively.

	*HL*	*HL* ^a^	*HL* ^b^	4*up*	4*dn*	**5**	
E_LUMO_ (-EA)	−1.881	−1.948	−2.111	−1.083	−1.714	−9.403	**4 ** *up > * **4 ** *dn > HL > HL* ^*a*^ * > HL* ^*b*^ *> * **5**
E_HOMO_ (-IP)	−5.653	−5.654	−5.808	−3.019	−3.229	−10.485	**4 ** *up > * **4 ** *dn > HL > HL* ^*a*^ * > HL* ^*b*^ *> * **5**
E_g_ = (E_LUMO_-E_HOMO_)	3.771	3.707	3.697	1.936	1.515	1.082	*HL > HL* ^*a*^ * > HL* ^*b*^ **4 ** *up. * **4 ** *dn > * **5**
*ƛ* = -(E_HOMO_ + E_LUMO_)/2	3.767	3.801	3.9591	2.051	2.4712	9.944	**5 ** * > HL* ^*b*^ * > HL* ^*a*^ * > H > * **4 ** *dn > * **4 ** *up*
*η* = (E_LUMO_-E_HOMO_)/2	1.886	1.853	1.849	0.968	0.757	0.541	*HL > HL* ^*a*^ * > HL* ^*b*^ *> * **4 ** *up > * **4 ** *dn > * **5**
*μ* = (E_HOMO_ + E_LUMO_)/2	−3.767	−3.801	−3.959	−2.051	−2.471	−9.944	**4 ** *up > * **4 ** *dn > HL > HL* ^*a*^ *<HL* ^*b*^ *> * **5**
*ω* = *μ*^2^/2 *η*	3.763	3.898	4.24	2.173	4.032	91.41	**5 ** * > HL* ^*b*^ *> * **4 ** *dn > HL* ^*a*^ * > HL > * **4 ** *up*

Antiproliferative activities, *HL*>**4** > **5**, Antibacterial, antifungal activities **5**>*HL* > **4**, Antiradical activity, **4**>(*HL*,*HL*^a^,*HL*^b^) **>** **5**.

## Data Availability

The data that supports the findings of this study are available in the supplementary material of this article.

## Supplementary Materials

Supplementary Materials CCDC 2129910–2129914 contains the supplementary crystallographic data for *HL*, *HL*^a^, *HL*^b^, and complexes **4** and **5**. Copies of the data can be obtained free of charge on application to CCDC, 12 Union Road, Cambridge CB2 1EZ, UK (fax: 44-1223-336–033; e-mail:deposit@ccdc.cam.ac.uk or www:http://www.ccdc.cam.ac.uk. Supplementary materials. Figure S1. 1H- NMR spectrum of 2-formylpyridine *N*^4^-allylthiosemicarbazone (*HL*).Figure S2. ^13^C- NMR spectrum of 2-formylpyridine *N*^4^-allylthiosemicarbazone (*HL*).Figure S3. 1H- NMR spectrum of 3-formylpyridine *N*^4^-allylthiosemicarbazone (*HL*^a^). Figure S4. ^13^C- NMR spectrum of 3-formylpyridine *N*^4^-allylthiosemicarbazone (*HL*^a^). Figure S5. ^1^H- NMR spectrum of 4-formylpyridine *N*^4^-allylthiosemicarbazone (*HL*^b^). Figure S6. ^13^C- NMR spectrum of 4-formylpyridine *N*^4^-allylthiosemicarbazone (*HL*^b^). Figure S7. UV-Vis spectra of the free ligand *HL* and complexes **1**–**6** in DMSO solutions. Figure S8. 9.5 GHz EPR spectra of complexes **2**, at 100 *μ*M (a), 200 *μ*M (b) and 500 *μ*M (c). Black dotted lines remind the position of the hyperfine lines corresponding to the Cu(II) complex with the ancillary ligand and a solvent molecule; red plain lines indicates the three first hyperfines lines corresponding to complex 2 where the c*hl*oride exogenous ligand stays bound. Table S1. Selected bond lengths in *HL*^a^ and *HL*^b^. Table S2. Hydrogen Bond Distances (Å) and Angles (deg) in *HL*, *HL*^a^, *HL*^b^, **4** and **5**.

## References

[1] Pahonțu E., Fala V., Gulea A., Poirier D., Tapcov V., Rosu T. (2013). Synthesis and characterization of some new Cu (II), Ni (II) and Zn (II) complexes with salicylidene thiosemicarbazones: antibacterial, antifungal and in vitro antileukemia activity. *Molecules*.

[2] Fischer C., Koenig B. (2011). Palladium- and copper-mediated N-aryl bond formation reactions for the synthesis of biological active compounds. *Beilstein Journal of Organic Chemistry*.

[3] Rosu T., Negoiu M., Pasculescu S., Pahontu E., Poirier D., Gulea A. (2010). Metal-based biologically active agents: synthesis, characterization, antibacterial and antileukemia activity evaluation of Cu(II), V(IV) and Ni(II) complexes with antipyrine-derived compounds. *European Journal of Medicinal Chemistry*.

[4] Ammaji S., Masthanamma S., Bhandare R. R., Annadurai S., Shaik A. B. (2022). Antitubercular and antioxidant activities of hydroxy and c*hl*oro substituted chalcone analogues: synthesis, biological and computational studies. *Arabian Journal of Chemistry*.

[5] Braña M., Cacho M., Ramos A. (2003). Synthesis, biological evaluation and DNA binding properties of novel mono and bisnaphthalimides. *Organic and Biomolecular Chemistry*.

[6] Graur V., Usataia I., Bourosh P. (2021). Synthesis, characterization, and biological activity of novel 3d metal coordination compounds with 2-acetylpyridine *N*^4^-allyl-S-methylisothiosemicarbazone. *Applied Organometallic Chemistry*.

[7] (2020). *WHO Report on Cancer: Setting Priorities, Investing Wisely and Providing Care for All*.

[8] de Santana T., de Oliveira Barbosa M., de Moraes Gomes P., da Cruz A., da Silva T., Leite A. (2018). Synthesis, anticancer activity and mechanism of action of new thiazole derivatives. *European Journal of Medicinal Chemistry*.

[9] Yadav U. P., Ansari A. J., Arora S. (2022). Design, synthesis and anticancer activity of 2-arylimidazo[1,2-a]pyridinyl-3-amines. *Bioorganic Chemistry*.

[10] Chabner B. A., Roberts T. G. (2005). Chemotherapy and the war on cancer. *Nature Reviews Cancer*.

[11] Pahontu E., Julea F., Rosu T. (2015). Antibacterial, antifungal and in vitro antileukaemia activity of metal complexes with thiosemicarbazones. *Journal of Cellular and Molecular Medicine*.

[12] Wang J., Zhang Z.-M., Li M.-X. (2022). Synthesis, characterization, and biological activity of cadmium (II) and antimony (III) complexes based on 2-acetylpyrazine thiosemicarbazones. *Inorganica Chimica Acta*.

[13] Belicchi Ferrari M., Bisceglie F., Pelosi G., Tarasconi P., Albertini R., Pinelli S. (2001). New methyl pyruvate thiosemicarbazones and their copper and zinc complexes: synthesis, characterization, X-ray structures and biological activity. *Journal of Inorganic Biochemistry*.

[14] Gulea A., Poirier D., Roy J. (2008). In vitro antileukemia, antibacterial and antifungal activities of some 3d metal complexes: chemical synthesis and structure-activity relationships. *Journal of Enzyme Inhibition and Medicinal Chemistry*.

[15] Pahonțu E., Paraschivescu C., Ilieș D. C. (2016). Synthesis and characterization of novel Cu(II), Pd(II) and Pt(II) complexes with 8-ethyl-2-hydroxytricyclo (7.3.1.02,7) tridecan-13-onethiosemicarbazone: antimicrobial and in vitro antiproliferative activity. *Molecules*.

[16] Manowitz M., Walter G. (1965). Antimicrobial properties of thiosemicarbazones of aliphatic ketones. *Journal of Pharmaceutical Sciences*.

[17] Gingras B. A., Colin G., Bayley C. H. (1965). Antifungal activity of thiosemicarbazones. *Journal of Pharmaceutical Sciences*.

[18] Carlsson F. H. H., Charlson A. J., Watton E. C. (1974). The biological activity of some guanylhydrazones and thiosemicarbazones of aliphatic carbonyl compounds. *Carbohydrate Research*.

[19] Kaplancıklı Z. A., Altıntop M. D., Sever B., Cantürk Z., Özdemir A. (2016). Synthesis and *In Vitro* evaluation of new thiosemicarbazone derivatives as potential antimicrobial agents. *Journal of Chemistry*.

[20] Karaküçük-İyidoğan A., Aydınöz B., Taşkın-Tok T., Oruç-Emre E. E., Balzarini J. (2019). Synthesis, biological evaluation and ligand based pharmacophore modeling of new aromatic thiosemicarbazones as potential anticancer agents. *Pharmaceutical Chemistry Journal*.

[21] Fatondji H. R., Kpoviessi S., Bero J. (2011). Synthesis, characterization and trypanocidal activity of 1,3,4- thiadiazolines derivatives. *African Journal of Pure and Applied Chemistry*.

[22] French F. A., Blanz E. J. (1966). The carcinostatic activity of thiosemicarbazones of formyl heteroaromatic Compounds. 3. primary correlation. *Journal of Medicinal Chemistry*.

[23] Booth B. A., Moore E. C., Sartorelli A. C. (1971). Metabolic effects of some tumor-inhibitory pyridine carboxaldehyde thiosemicarbazones. *Cancer Research*.

[24] Easmon J., Heinisch G., Holzer W., Rosenwirth B. (1989). Synthesis and antiviral activity of thiosemicarbazone derivatives of pyridazinecarbaldehydes and alkyl pyridazinyl ketones. *Arzneimittel Forschung*.

[25] Matesanz A., Souza P. (2009). Alpha-N-heterocyclic thiosemicarbazone derivatives as potential antitumor agents: a structure-activity relationships approach. *Mini Reviews in Medicinal Chemistry*.

[26] Agrawal K. C., Sartorelli A. C. (1978). The chemistry and biological activity of *α*-(N)-heterocyclic carboxaldehyde thiosemicarbazones. *Progress in Medicinal Chemistry*.

[27] Beraldo H., Gambino D. (2004). The wide pharmacological versatility of semicarbazones, thiosemicarbazones and their metal complexes. *Mini Reviews in Medicinal Chemistry*.

[28] Richardson D. R., Kalinowski D. S., Richardson V. (2009). 2-acetylpyridine thiosemicarbazones are potent iron chelators and antiproliferative agents: redox activity, iron complexation and characterization of their antitumor activity. *Journal of Medicinal Chemistry*.

[29] Knox J. J., Hotte S. J., Kollmannsberger C., Winquist E., Fisher B., Eisenhauer E. A. (2007). Phase II study of triapine in patients with metastatic renal cell carcinoma: a trial of the national cancer institute of Canada clinical trials group (NCIC IND.161). *Investigational New Drugs*.

[30] Stacy A. E., Palanimuthu D., Bernhardt P. V., Kalinowski D. S., Jansson P. J., Richardson D. R. (2016). Structure-activity relationships of di-2-pyridylketone, 2-benzoylpyridine, and 2-acetylpyridine thiosemicarbazones for overcoming Pgp-mediated drug resistance. *Journal of Medicinal Chemistry*.

[31] Agarwal R. K., Singh L., Sharma D. K. (2006). Synthesis, biological, spectral, and thermal investigations of cobalt(II) and nickel(II) complexes of N-isonicotinamido-2′,4′-dic*hl*orobenzalaldimine. *Bioinorganic Chemistry and Applications*.

[32] Pósa V., Hajdu B., Tóth G. (2022). The coordination modes of (thio)semicarbazone copper(II) complexes strongly modulate the solution chemical properties and mechanism of anticancer activity. *Journal of Inorganic Biochemistry*.

[33] Hegde P. L., Bhat S. S., Revankar V. K., Shaikh S. A., Kumara K., Lokanath N. K. (2022). Syntheses, structural characterization and evaluation of the anti-tubercular activity of copper (II) complexes containing 3-methoxysalicylaldehyde-4-methylthiosemicarbazone. *Journal of Molecular Structure*.

[34] Ferrari M. B., Fava G. G., Tarasconi P., Albertini R., Pinelli S., Starcich R. (1994). Synthesis, spectroscopic and structural characterization, and biological activity of aquac*hl*oro(pyridoxal thiosemicarbazone) copper(II) c*hl*oride. *Journal of Inorganic Biochemistry*.

[35] Drzewiecka-Antonik A., Rejmak P., Klepka M., Wolska A., Chrzanowska A., Struga M. (2020). Structure and anticancer activity of Cu(II) complexes with (bromophenyl)thiourea moiety attached to the polycyclic imide. *Journal of Inorganic Biochemistry*.

[36] Pulvermacher G. (1894). Ueber einige abkömmlinge des thiosemicarbazids und umsetzungsproducte derselben. *Berichte der Deutschen Chemischen Gesellschaft*.

[37] Zhao W., Zhao M. (2001). Synthesis and characterization of some multi-substituted thiosemicarbazones as the multi-dental ligands of mtal ions. *Chinese Journal of Organic Chemistry*.

[38] Perrin D. D., Armarego W. L., Perrin D. R. (1990). *Purification of Laboratory Chemicals*.

[39] Zeglis B. M., Divilov V., Lewis J. S. (2011). Role of metalation in the topoisomerase II*α* inhibition and antiproliferation activity of a series of *α*-heterocyclic-N^4^-substituted thiosemicarbazones and their Cu(II) complexes. *Journal of Medicinal Chemistry*.

[40] Fries J., Getrost H. (1977). *Organic Reagents for Trace Analysis*.

[41] Sheldrick G. M. (2008). A short history of SHELX. *Acta Crystallographica Section A Foundations of Crystallography*.

[42] Sheldrick G. M. (2015). Crystal structure refinement with SHELXL. *Acta Crystallographica*.

[43] Spek A. L. (2009). Structure validation in chemical crystallography. *Acta Crystallographica Section D Biological Crystallography*.

[44] Macrae C. F., Sovago I., Cottrell S. J. (2020). Mercury 4.0: from visualization to analysis, design and prediction. *Journal of Applied Crystallography*.

[45] Balan G., Burduniuc O., Usataia I. (2020). *Applied Organometallic Chemistry*.

[46] Re R., Pellegrini N., Proteggente A., Pannala A., Yang M., Rice-Evans C. (1999). Antioxidant activity applying an improved ABTS radical cation decolorization assay. *Free Radical Biology and Medicine*.

[47] Ferraz K. O., Wardell S. M. S. V., Wardell J. L., Louro S. R. W., Beraldo H. (2009). Copper(II) complexes with 2-pyridineformamide-derived thiosemicarbazones: spectral studies and toxicity against *Artemia salina*. *Spectrochimica Acta Part A: Molecular and Biomolecular Spectroscopy*.

[48] Alaghaz A.-N. M. A., El-Sayed B. A., El-Henawy A. A., Ammar R. A. A. (2013). Synthesis, spectroscopic characterization, potentiometric studies, cytotoxic studies and molecular docking studies of DNA binding of transition metal complexes with 1,1-diaminopropane-schiff base. *Journal of Molecular Structure*.

[49] Wilkinson G., Gillard R. D., McCleverty J. A. (1987). *Comprehensive Coordination Chemistry*.

[50] Hay R. W. (2000). *Reaction Mechanisms of Metal Complexes*.

[51] Al-Amiery A. A., Kadhum A. A. H., Mohamad A. B. (2012). Antifungal and antioxidant activities of pyrrolidone thiosemicarbazone complexes. *Bioinorganic Chemistry and Applications*.

[52] Lever A. P. B. (1984). *Inorganic Electronic Spectroscopy*.

[53] Peisach J., Blumberg W. E. (1974). Structural implications derived from the analysis of electron paramagnetic resonance spectra of natural and artificial copper proteins. *Archives of Biochemistry and Biophysics*.

[54] Ufnalska I., Drew S. C., Zhukov I. (2021). Intermediate Cu(II)-thiolate species in the reduction of Cu(II)GHK by glutathione: a handy chelate for biological Cu(II) reduction. *Inorganic Chemistry*.

[55] Alibek K., Bekmurzayeva A., Mussabekova A., Sultankulov B. (2012). Using antimicrobial adjuvant therapy in cancer treatment: a review. *Infectious Agents and Cancer*.

[56] Kardas J., Buraczewska A. (2016). Maintenance chemotherapy with pemetrexed in patients with malignant pleural mesothelioma-case series and review of the literature. *Oncology in Clinical Practice*.

[57] Zhaleh M., Zangeneh A., Goorani S. (2019). In vitro and in vivo evaluation of cytotoxicity, antioxidant, antibacterial, antifungal, and cutaneous wound healing properties of gold nanoparticles produced via a green chemistry synthesis using Gundelia tournefortii L. as a capping and reducing agent. *Applied Organometallic Chemistry*.

[58] Gulea А. P., Graur V. О., Ulchina I. I. (2021). Synthesis, structure, and biological activity of mixed-ligand amine-containing copper(II) coordination compounds with 2-(2-hydroxybenzylidene)-N-(prop-2-en-1-yl)hydrazinecarbothioamide. *Russian Journal of General Chemistry*.

[59] Frisch M., Trucks G. W., Schlegel H. B. (2016). *Gaussian 16 Revision C*.

[60] Lu T., Chen F. (2012). Multiwfn: a multifunctional wavefunction analyzer. *Journal of Computational Chemistry*.

[61] Gatfaoui S., Issaoui N., Brandán S. A., Roisnel T., Marouani H. (2018). Synthesis and characterization of p-xylylenediaminium bis(nitrate). effects of the coordination modes of nitrate groups on their structural and vibrational properties. *Journal of Molecular Structure*.

[62] Politzer P., Murray J. S., Beveridge D. L., Lavery R. (1991). *Theoretical Biochemistry and Molecular Biophysics: A Comprehensive Survey. Protein*.

[63] Scrocco E., Tomasi J. (1973). *Current Chemistry*.

[64] Chakraborty A., Pan S., Chattaraj P. K. (2013). Biological activity and toxicity: a conceptual DFT approach. *Structure and Bonding*.

[65] Parr R. G., Pearson R. G. (1983). Absolute hardness: companion parameter to absolute electronegativity. *Journal of the American Chemical Society*.

[66] Irfan A., Imran M., Al-Sehemi A. G., Shah A. T., Hussien M., Mumtaz M. W. (2021). Exploration of electronic properties, radical scavenging activity and QSAR of oxadiazole derivatives by molecular docking and first-principles approaches. *Saudi Journal of Biological Sciences*.

[67] Noureddine O., Issaoui N., Gatfaoui S., Al-Dossary O., Marouani H. (2021). Quantum chemical calculations, spectroscopic properties and molecular docking studies of a novel piperazine derivative. *Journal of King Saud University Science*.

[68] Ishiguro K., Lin Z. P., Penketh P. G. (2014). Distinct mechanisms of cell-kill by triapine and its terminally dimethylated derivative Dp44mT due to a loss or gain of activity of their copper(II) complexes. *Biochemical Pharmacology*.

