# Supplementary material for: Synthesis, Structure, and Biologic Activity of Some Copper, Nickel, Cobalt, and Zinc Complexes with 2-Formylpyridine N4-Allylthiosemicarbazone
Source: Bioinorg Chem Appl. 2022 May 25;2022:2705332. doi: 10.1155/2022/2705332 (PMC9159852; doi:10.1155/2022/2705332)
Supplement: Supplementary Materials — CCDC 2129910–2129914 contains the supplementary crystallographic data for HL, HLa, HLb, and complexes 4 and 5. Copies of the data can be obtained free of charge on application to CCDC, 12 Union Road, Cambridge CB2 1EZ, UK (fax: 44-1223-336–033; e-mail:deposit@ccdc.cam.ac.uk or www:http://www.ccdc.cam.ac.uk. Supplementary materials. Figure S1. 1H- NMR spectrum of 2-formylpyridine N4-allylthiosemicarbazone (HL).Figure S2. 13C- NMR spectrum of 2-formylpyridine N4-allylthiosemicarbazone (HL).Figure S3. 1H- NMR spectrum of 3-formylpyridine N4-allylthiosemicarbazone (HLa). Figure S4. 13C- NMR spectrum of 3-formylpyridine N4-allylthiosemicarbazone (HLa). Figure S5. 1H- NMR spectrum of 4-formylpyridine N4-allylthiosemicarbazone (HLb). Figure S6. 13C- NMR spectrum of 4-formylpyridine N4-allylthiosemicarbazone (HLb). Figure S7. UV-Vis spectra of the free ligand HL and complexes 1–6 in DMSO solutions. Figure S8. 9.5 GHz EPR spectra of complexes 2, at 100 μM (a), 200 μM (b) and 500 μM (c). Black dotted lines remind the position of the hyperfine lines corresponding to the Cu(II) complex with the ancillary ligand and a solvent molecule; red plain lines indicates the three first hyperfines lines corresponding to complex 2 where the chloride exogenous ligand stays bound. Table S1. Selected bond lengths in HLa and HLb. Table S2. Hydrogen Bond Distances (Å) and Angles (deg) in HL, HLa, HLb, 4 and 5. [file 2705332.f1.docx]

Supplementary materials


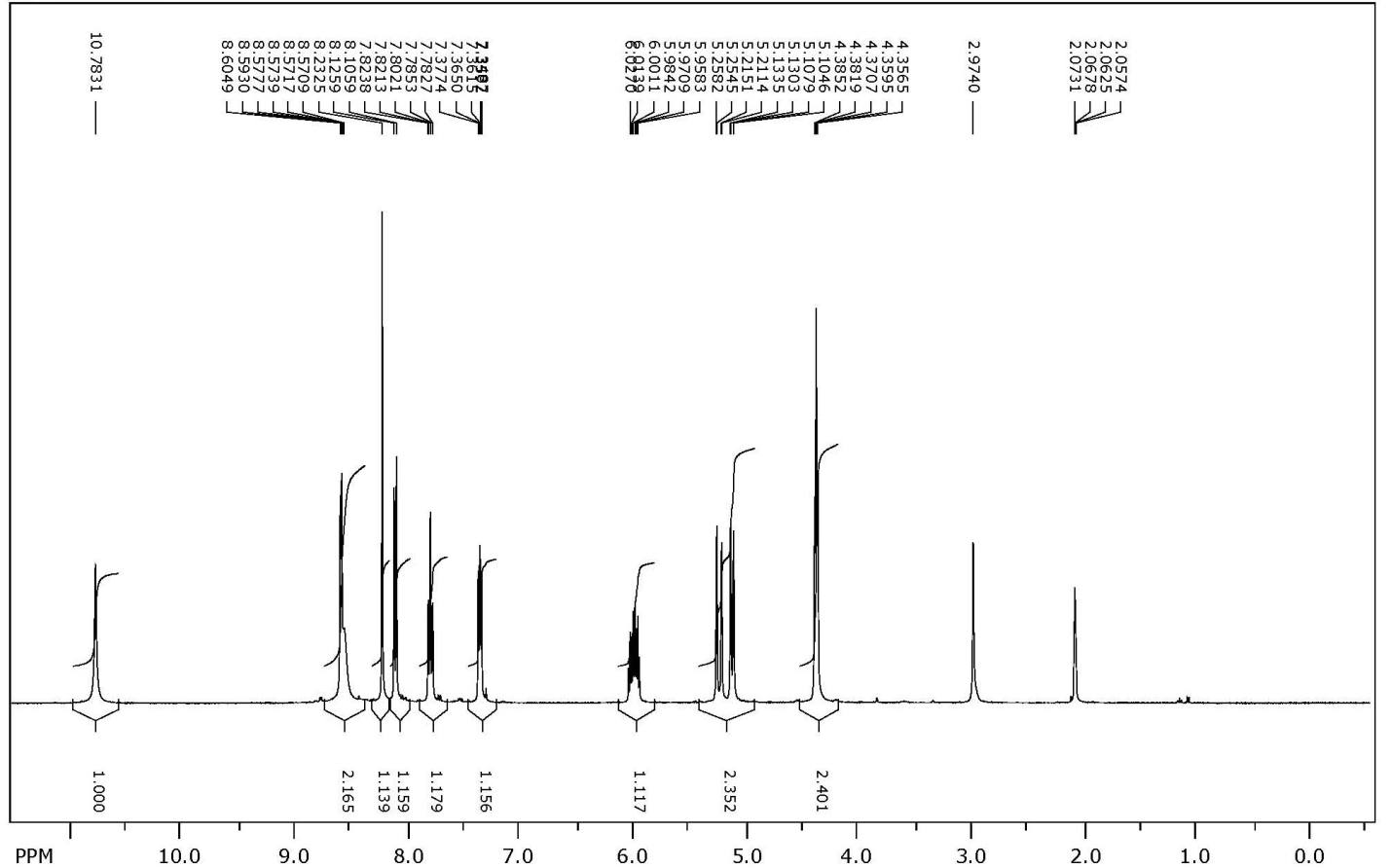


**Figure S1**. ^1^H- NMR spectrum of 2-formylpyridine *N*^4^-allylthiosemicarbazone (**HL**).


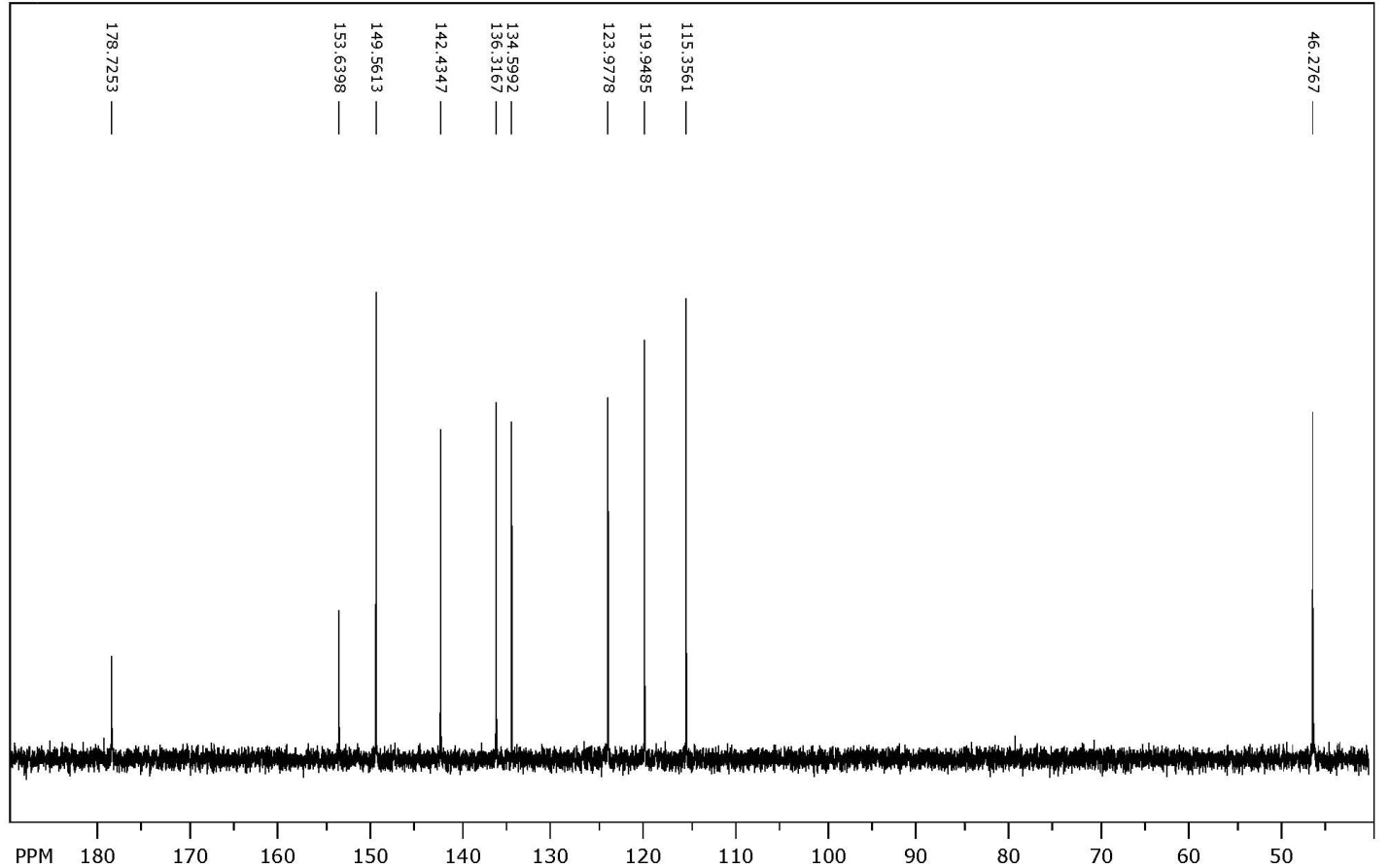


**Figure S2**. ^13^C- NMR spectrum of 2-formylpyridine *N*^4^-allylthiosemicarbazone (**HL**).


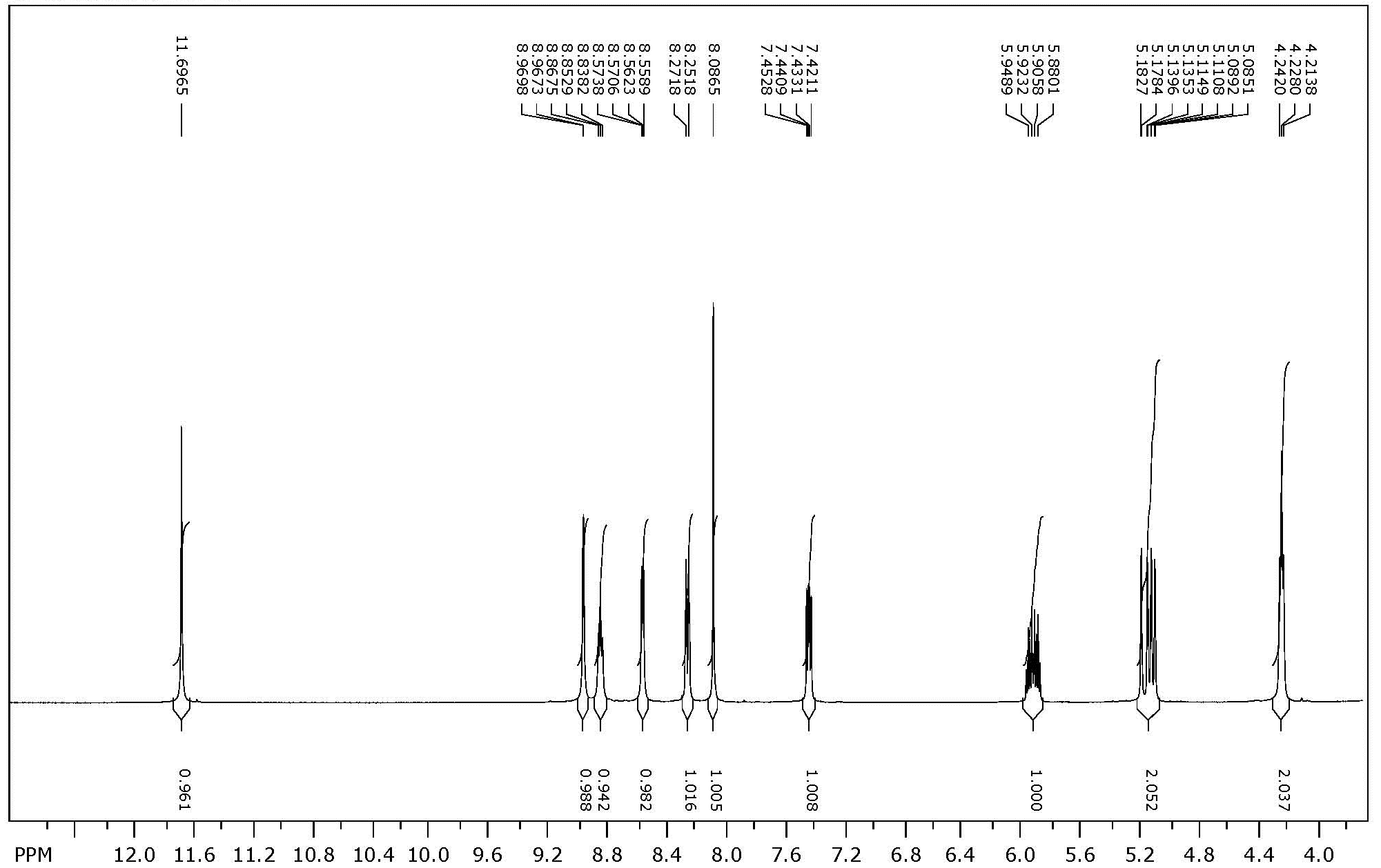


**Figure S3**. ^1^H- NMR spectrum of 3-formylpyridine *N*^4^-allylthiosemicarbazone (**HL^a^**).


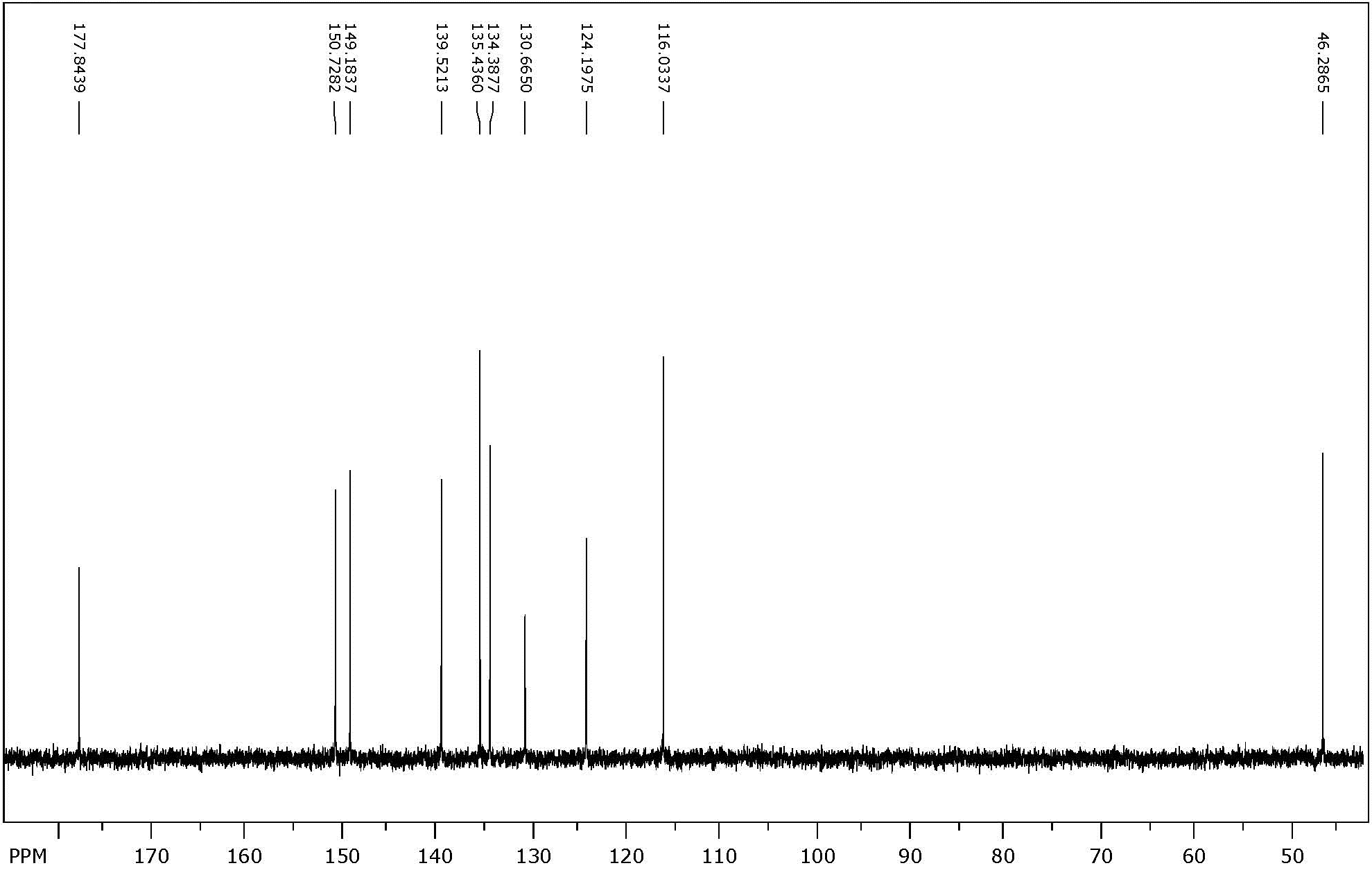


**Figure S4**. ^13^C- NMR spectrum of 3-formylpyridine *N*^4^-allylthiosemicarbazone (**HL^a^**).


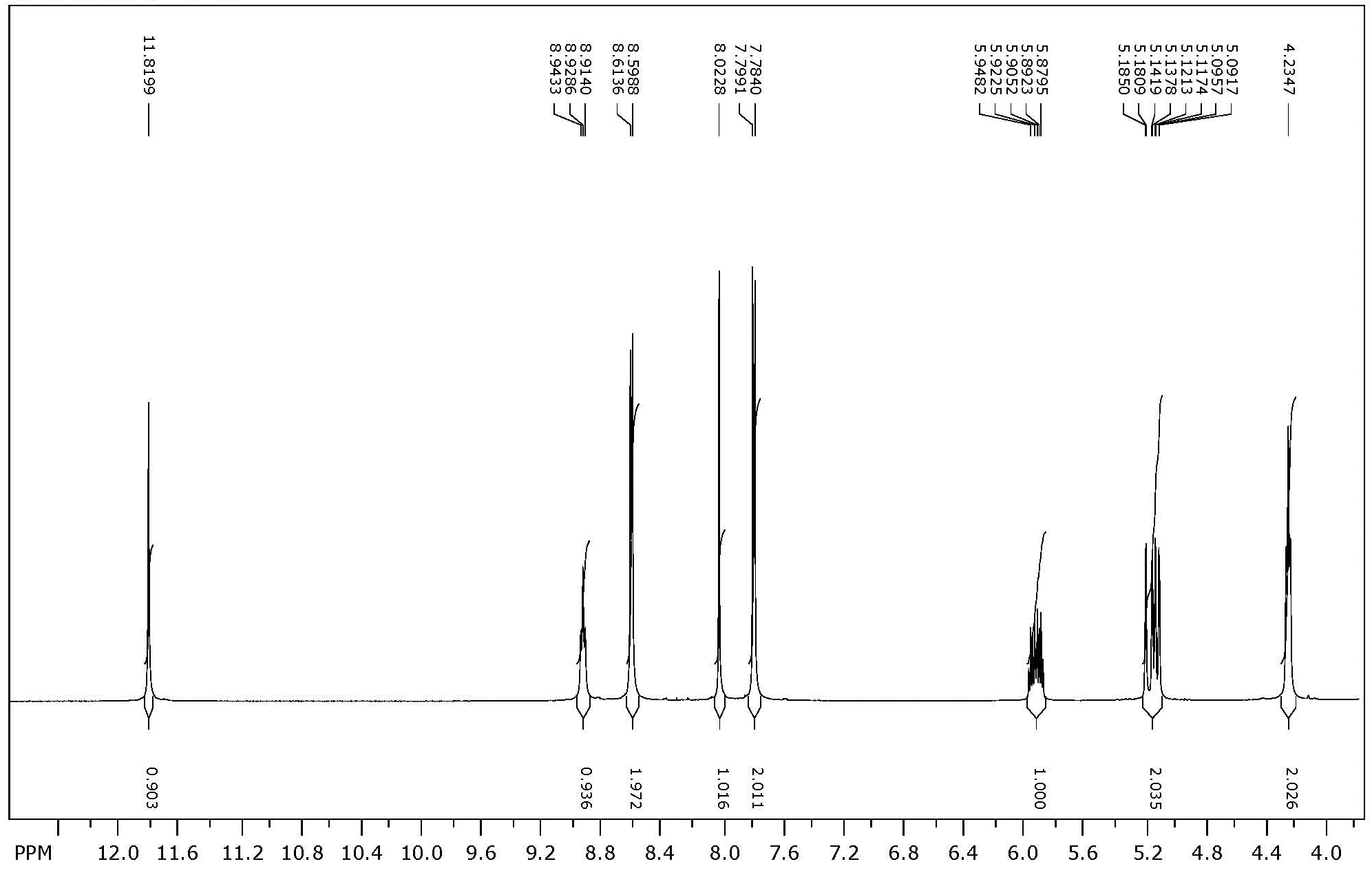


**Figure S5**. ^1^H- NMR spectrum of 4-formylpyridine *N*^4^-allylthiosemicarbazone (**HL^b^**).


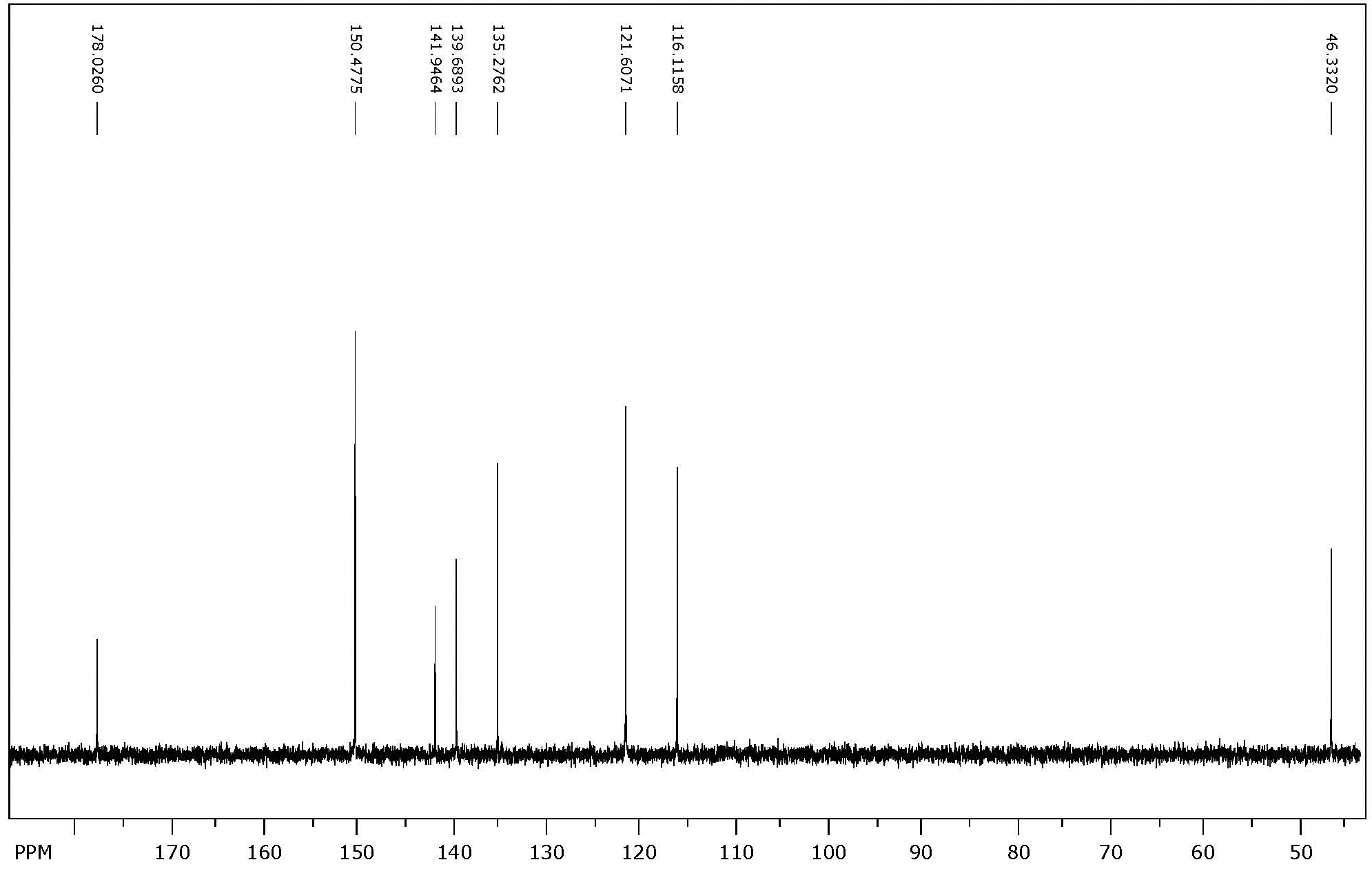


**Figure S6**. ^13^C- NMR spectrum of 4-formylpyridine *N*^4^-allylthiosemicarbazone (**HL^b^**).


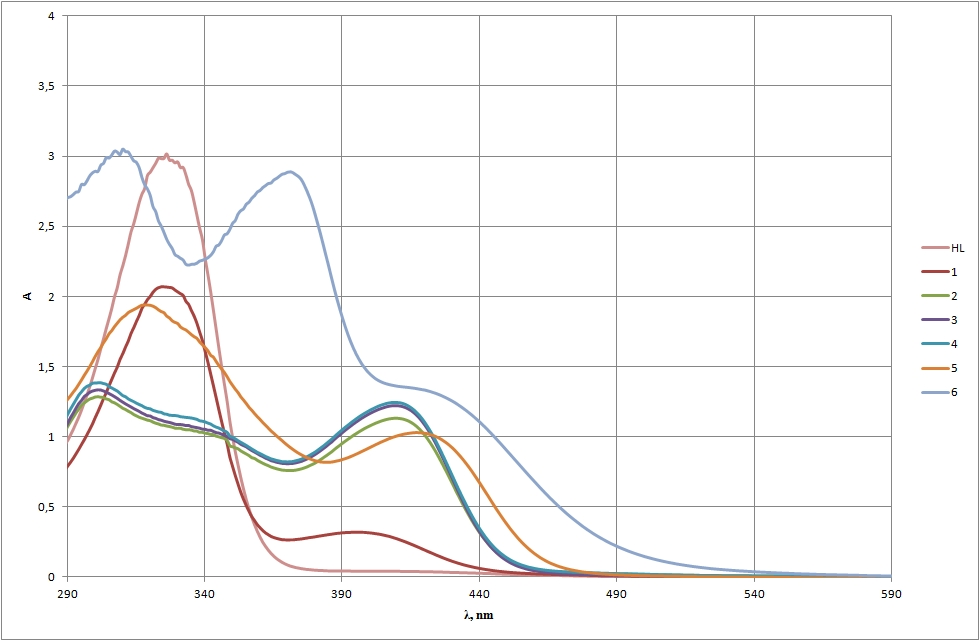


**Figure S7**. UV-Vis spectra of the free ligand **HL** and complexes **1** - **6** in DMSO solutions.


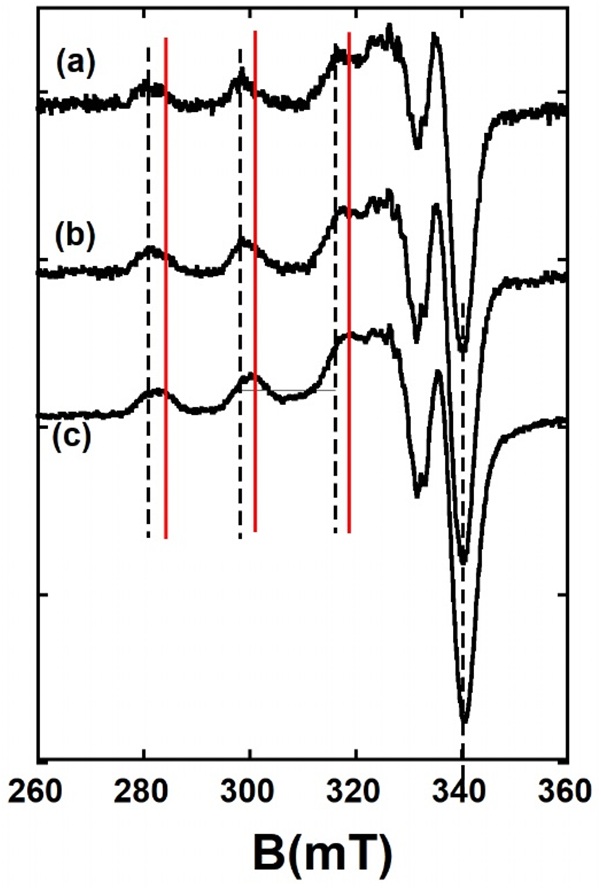


**Figure S8.** 9.5 GHz EPR spectra of complexes **2**, at 100 µM (a), 200 µM (b) and 500 µM (c). Black dotted lines remind the position of the hyperfine lines corresponding to the Cu(II) complex with the ancillary ligand and a solvent molecule; red plain lines indicates the three first hyperfines lines corresponding to complex **2** where the chloride exogenous ligand stays bound.

Table S1. Selected bond lengths in **HL^a^** and **HL^b^.**

| Bond | **HL^a^** | **HL^b^** | | |
| --- | --- | --- | --- | --- |
| S1-C1 | 1.680(3) | 1.680(3) | 1.684(3) | 1.684(3) |
| N1-N2 | 1.365(3) | 1.365(3) | 1.373(3) | 1.374(3) |
| N1-C2 | 1.279(4) | 1.279(4) | 1.27(4) | 1.276(4) |
| N2-C1 | 1.365(3) | 1.365(3) | 1.373(3) | 1.355(4) |
| C2-C3 | 1.454(4) | 1.454(4) | 1.451(4) | 1.458(4) |

Table S2. Hydrogen Bond Distances (Å) and Angles (deg) in **HL**, **HL^a^**, **HL^b^**, **4** and **5**

| D–H⋅⋅⋅A | d(H⋅⋅⋅A) | d(D⋅⋅⋅A) | ∠(DHA) | Symmetry transformation for acceptor |
| --- | --- | --- | --- | --- |
| **HL** | | | | |
| N2-H...N4 | 2.15 | 3.012(5) | 175.0 | -x,-1/2+y,1-z |
| **HL^a^** | | | | |
| N2-H... N4 | 2.13 | 2.975(6) | 166.0 | -1/2+x,1/2+y,z |
| **HL^b^** | | | | |
| N2A-H... S1A | 2.53 | 3.370(2) | 167.0 | -x,1-y,-z |
| N3-H... N4B | 2.27 | 3.039(4) | 148.0 | x,3/2-y,-1/2+z |
| N3A-H... N4A | 2.21 | 2.972(3) | 147.0 | -x,1/2+y,1/2-z |
| N3B-H... N4 | 2.24 | 2.984(4) | 145.0 | x,1/2-y,1/2+z |
| **4** | | | | |
| O1W-H... O2W | 2.14 | 2.822(4) | 173.0 | x,y,z |
| O2W-H... N2 | 2.45 | 3.123(6) | 149.0 | -1+x,y,z |
| O1W-H1... O2 | 1.72 | 2.714(4) | 177.0 | 1-x,-y,1-z |
| N3-H... O1W | 2.12 | 2.924(4) | 155.0 | x,y,-1+z |
| O2W-H... O1W | 2.05 | 2.842(5) | 167.0 | x,1/2-y,-1/2+z |
| C2-H... O2 | 2.48 | 3.239(4) | 139.0 | 2-x,-y,1-z |
| **5** | | | | |
| N2A-H... Cl1 | 2.21 | 3.015(4) | 156.0 | -x,1-y,1-z |
| N3A-H... Cl1 | 2.46 | 3.241(4 | 151.0 | -x,1-y,1-z |
| N3-H... Cl2 | 2.44 | 3.233(4) | 153.0 | 1-x,1-y,2-z |
| N2-H... Cl2 | 2.32 | 3.120(4) | 155.0 | 1-x,1-y,2-z |
